# One-step high-speed thermal-electric aerosol printing of piezoelectric bio-organic films for wirelessly powering bioelectronics

**DOI:** 10.1126/sciadv.adq3195

**Published:** 2024-10-25

**Authors:** Xuemu Li, Zhuomin Zhang, Yi Zheng, Junchen Liao, Zehua Peng, Pengyu Li, Xiaodan Yang, Xiaodong Yan, Ying Hong, Shiyuan Liu, Yao Shan, Bee Luan Khoo, Zhengbao Yang

**Affiliations:** ^1^ Department of Mechanical and Aerospace Engineering, Hong Kong University of Science and Technology, Clear Water Bay, Hong Kong, China.; ^2^ Department of Mechanical Engineering, City University of Hong Kong, Hong Kong, China.; ^3^ Department of Biomedical Engineering, City University of Hong Kong, Hong Kong, China.; ^4^ Institute of Electrical and Micro Engineering, École Polytechnique Fédérale de Lausanne, 1015 Lausanne, Switzerland.

## Abstract

Piezoelectric biomaterials hold a pivotal role in the progression of bioelectronics and biomedicine, owing to their remarkable electromechanical properties, biocompatibility, and bioresorbability. However, their technological potential is restrained by certain challenges, including precise manipulation of nanobiomolecules, controlling their growth across nano-to-macro hierarchy, and tuning desirable mechanical properties. We report a high-speed thermal-electric driven aerosol (TEA) printing method capable of fabricating piezoelectric biofilms in a singular step. Electrohydrodynamic aerosolizing and in situ electrical poling allow instantaneous tuning of the spatial organization of biomolecular inks. We demonstrate TEA printing of β-glycine/polyvinylpyrrolidone films, and such films exhibit the piezoelectric voltage coefficient of 190 × 10^−3^ volt-meters per newton, surpassing that of industry-standard lead zirconate titanate by approximately 10-fold. Furthermore, these films demonstrate nearly two orders of magnitude improvement in mechanical flexibility compared to glycine crystals. We also demonstrate the ultrasonic energy harvesters based on the biofilms, providing the possibility of wirelessly powering bioelectronics.

## Teaser

Flexible piezoelectric biomolecular films are fabricated with a one-step, high-speed strategy.

## INTRODUCTION

Since the discovery of piezoelectricity in single-crystal Rochelle salt in 1880 and its subsequent extension into the realm of polycrystalline ceramics and polymers (*1*–*3*), piezoelectric materials have been ubiquitous key components in scientific and technological innovations for micro-electronics, optics, acoustics, and energy-related systems (*4*–*9*). An ongoing surge in the demand for miniaturized/flexible bioelectronics, wearable/implantable microdevices, and biotissue therapeutics has inspired the relentless pursuit of developing biofriendly piezoelectric materials. Natural piezoelectric biomaterial is one such attribute that has superiority over the widely used ceramics and polymers owing to their excellent portability, biocompatibility, biodegradability, and low weight/cost (*10*–*12*). Since the first indication of polarization in wool and hair in 1941, piezoelectricity has been discovered in many biomaterials, as exemplified by skin, tendon, cartilage, ligament, cornea, sclera, wood, silk, chitin, and amino acids (*13*–*19*). However, it remains technically challenging for practical applications, because of their weak macroscopic piezoelectricity, poor mechanical properties, and the incapability of mass production. Piezoelectricity is considered the result of long-range ordering of internal molecular or ionic dipoles, and the electrical aligning or poling has been identified as the key to turning inert crystals into electromechanically active materials (*20*). Nevertheless, the intrinsic complex and hierarchical structures of various piezoelectric biomaterials (such as amino acids and peptides) exclude them from post-electrical poling. The aligning polarization process of the crystalline phase also leads to material rigidity, brittleness, and weak strain resistance (*21*–*23*). Several assembly strategies (*24*, *25*), based on external agent–added solution, vapors, and heterogeneous surfaces, have been proposed to fabricate biomolecular structures (*26*–*28*). However, these methods still suffer from challenges regarding cross-scale and programmed manufacturing, due to the time-consuming domain aligning process and the limited active control of biomolecule growth (*29*).

Transient implanted devices, such as temporary postoperative cardiac pacemakers, require percutaneous leads or implanted batteries (*30*). Such hardware introduces risks for systemic infections, constrains patient mobility, and impedes clinical care. Surgical procedures for the removal or replacement of these batteries also lead to further tissue damage. Hence, there has been a pursuit of biocompatible and biodegradable implanted powering systems to prevent these issues. Piezoelectric ultrasonic energy harvesters (PUEHs) that convert mechanical vibrations of ultrasonic waves into electrical energy using the piezoelectric effect have been developed for wirelessly powering implanted devices in vivo (*31*–*34*). The PUEHs have advantages, including noninvasive remote energy transfer, low attenuation through tissue, short wavelengths for more efficient coupling to miniaturized implants, and high security (*35*, *36*). Currently, PUEH receivers often consist of inorganic piezoelectric ceramics [such as lead zirconate titanate (PZT)], which are always intrinsically rigid, unbiodegradable, and may contain toxic elements. Even synthetic flexible piezoelectric polymers [for example, polyvinylidene fluoride (PVDF)] do not meet the requirement of biodegradability. These drawbacks exclude them from clinical applications in on-demand transient devices. A new generation of biocompatible, biodegradable, and flexible piezoelectric materials and related PUEHs need to be developed.

Here, we devise a thermal-electric aerosol (TEA) printing strategy, which leverages a thermal-electric field to control electrical poling and spatial assemblies of biomolecules on the fly and then fix a long-range ordering of molecules on the substrate. The instantaneous synergy of electrical poling and nanoscale spatial assemblies of biomolecules is the key to the TEA printing strategy, which is unobtainable in conventional methods using bulk solution based on liquid-solid interface or ultrahigh electric field (*37*–*39*). As a demonstration, we print the piezoelectric β-glycine/polyvinylpyrrolidone (glycine/PVP) films. These films exhibit exceptional properties, including a high piezoelectric voltage coefficient *g*_33_ of 190 × 10^−3^ Vm N^−1^ (one order of magnitude higher than that of the industry-standard piezoceramic, PZT), a high piezoelectric failure temperature of 185°C [65°C above that of barium titanate (BTO)], and nearly two orders of magnitude enhancement of the mechanical flexibility compared to glycine crystals. In terms of the developed glycine-based PUEHs, the stable wireless outputs (~35 μW cm^−1^) ensure their charging efficacy for transient implanted devices. The combination of top-down design freedom offered by additive manufacturing and bottom-up control over nanobiomolecules showcases the feasibility and boundless prospects in bridging the gap between laboratory piezoelectric biomaterials and practical biodevices.

## RESULTS AND DISCUSSION

### TEA printing strategy

**Fig. 1.**
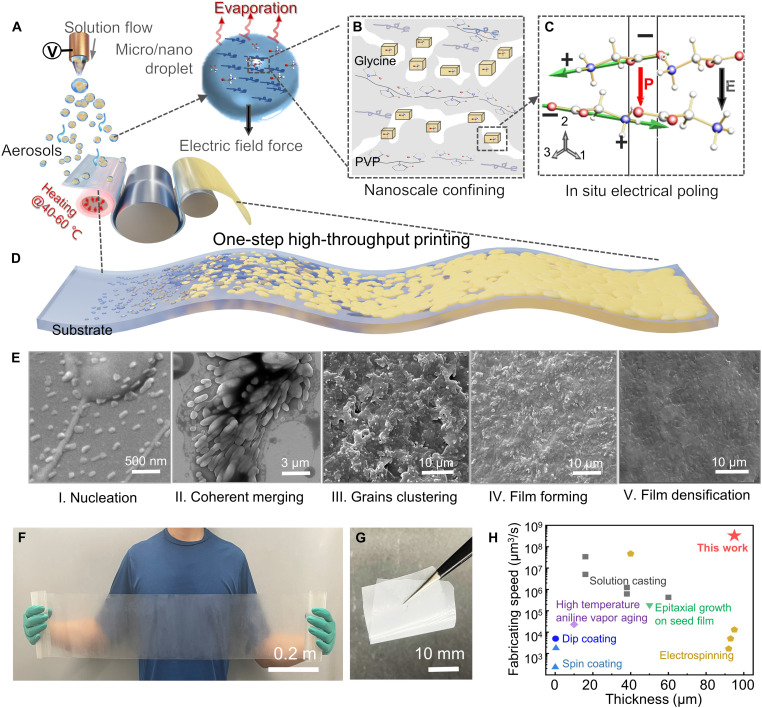
The TEA printing strategy. (**A**) Schematic illustration of the TEA method for fabricating natural piezoelectric biomaterials based on (**B**) nanoscale confining and (**C**) in situ aerosol poling. (**D**) Schematic illustration of the roll-based production of piezoelectric biofilms deposited a conductive foil. (**E**) SEM images of the structural evolution from scattered particles to clustered particles, and last, the fusing film. (**F**) Photograph of a roll of glycine/PVP films. (**G**) As-deposited glycine/PVP film showing outstanding flexibility. (**H**) Comparison of fabricating capabilities regarding manufacturing speed and the accessible film thickness of existing techniques with the TEA printing method.

We here present a TEA printing strategy to actively control the assembly process and in situ program biomolecules’ nano-to-macro growth. Figure 1 illustrates the TEA printing strategy. To achieve aerosol-based printing, we begin with electrohydrodynamic atomization of glycine/PVP composite inks into microscale droplets, where the droplets then undergo shrinking and splitting into nanodroplets, and electric dynamically dispersing (Fig. 1A). Once aerosolized, the geometrical dimensions of nanodroplets depend on the electrostatic repulsion and solvent evaporation. As the solvent from the atomized droplets evaporates, the nucleation of β-polymorph is favorable due to the nanoconfinement effect (Fig. 1B, fig. S1, and notes S1 and S2). The external (direct current) DC electric field can in situ manipulate the domain alignment of the in-flight crystals and induce the net polarization direction [020] parallel to the electric field (Fig. 1C). Flexible conductive foils can be used in the form of roll-type substrates fitting under multiple printing nozzles to maximize the printing scale (figs. S2 and S3, table S1, and movie S1). Thermally driven high supersaturating and rapid filming processes further allow efficient printing process (Fig. 1, A and D). The filming process can be described as five elementary stages: I, nucleation; II, coherent merging; III, grains clustering; IV, film forming; and V, densification (Fig. 1E). First, numerous nanocrystals enwrapped with polymer are deposited as isolated islands and are aligned in the polar direction [020]. The surface roughness then rapidly increases in the following island coherent merging, and granular protrusions appear. Last, films with defect-free and polymer-like smooth surfaces are formed (Fig. 1E and fig. S4). By facilely controlling the ink properties and printing parameters, large-scale and flexible glycine/PVP films are fabricated (Fig. 1, F and G, and fig. S5). Fourier transform infrared (FTIR) spectrum of glycine/PVP films indicate that the typical peaks (pyrrolidinyl group and C═O vibration) of PVP exist in the composite films, and these major chemical bonds remain unchanged (fig. S6A). The scanning electron microscopy (SEM) and transmission electron microscopy images show the packing of the glycine crystals inside the polymers (fig. S6, B and C).

Traditional biomolecular assembly methods often require extensive self-aligning time (from ~0.5 to ~48 hours), which not only brings difficulties for high-speed manufacturing but also leads to some undesired structural defects. By contrast, the TEA method can fabricate piezoelectric bio-organic films as thick as ~100 μm (Fig. 1H, fig. S7, and table S2) with high manufacturing speed up to 10^8^ μm^3^ s^−1^, two orders of magnitude faster than the existing techniques. Moreover, TEA printing uses electric field force to manipulate the aerosols, which can form electric repulsion between these aerosols such that the nucleation process will not be disturbed until deposition onto the substrates.

### Process optimization

**Fig. 2.**
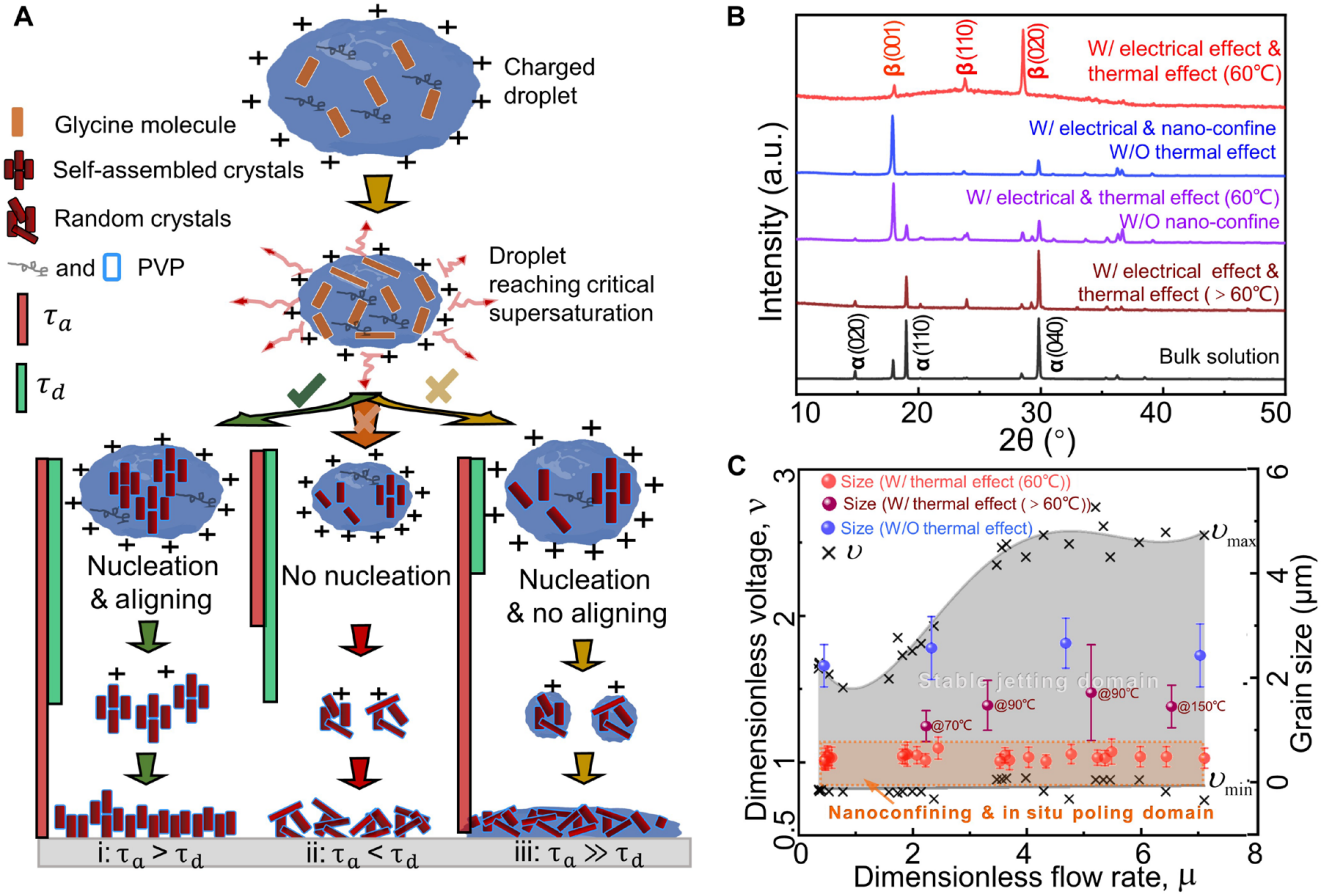
Process optimization of the TEA printing. (**A**) Mechanistic diagram of the TEA printing process based on the electrohydrodynamic aerosolized droplets. (**B**) XRD spectra of the as-deposited β-glycine/PVP films (red curve), glycine/PVP films obtained in the absence of thermal field (blue curve), glycine microcrystals fabricated by electrohydrodynamic focused printing (purple curve), α crystals formed in an excessive thermal field (dark red curve), and α crystals formed by direct evaporation of bulk glycine/PVP solution (black curve). W/ denotes with, and W/O denotes without. (**C**) Jetting maps in terms of the dimensionless flow rate and dimensionless voltage. This map enabled a quantitative description of the stable jetting mode in terms of the process parameters (gray area). The results of phase and grain size determined the nanoscale confining and in situ poling domain (orange area).

The desired printing of glycine/PVP films relies on two hypotheses: (i) nucleation of aerosols on the fly and (ii) in situ domain aligning. In the thermal-electric coupled field, the glycine/PVP ink at a desired initial concentration (*c_i_*) is atomized, and charged droplets with a diameter of *d_i_* are formed. With the increase of surface area–to–volume ratio of the atomized droplets, the increased concentration and supersaturation will drive the nucleation and domain aligning (Fig. 2A). The supersaturating ratio is

$$S\left(c_{i},d_{i}\right)=\frac{c-c_{e}}{c_{e}}=\frac{c_{i}}{c_{e}{\left(1-T/T_{e}\right)}^{3/2}}-1$$ (1)

where *T*_e_(*d_i_*) = (*R*ρ_d_ *d2 it*_d_)/(8*D*_v_*MP*_d_) is the evaporation time of droplets, *R* is the universal gas constant (8.314 J K^−1^ mol^−1^), ρ_d_ is the droplet density, *t*_d_ is the temperature, and *c*_e_ is the equilibrium concentration of the monomer. *D*_v_, *M*, and *P*_d_ are the diffusion coefficient, molecular weight, and vapor pressure of ink, respectively. Nucleation tends to occur when the supersaturation ratio of the droplet reaches the critical supersaturation ratio (S^*^), and this process only occurs within a finite time after critical supersaturation

$$T_{d}\left(c_{i},d_{i}\right)=\frac{32}{\pi^{2}}\frac{v_{0}\sigma^{2}}{\alpha^{*}{\left({\mathrm{kt}}_{d}\right)}^{2}{\mathrm{DNc}}_{e}\left(S+1\right){\text{ln}}^{3}\left(S+1\right)}$$ (2)

where α^*^ is the accommodation coefficient of ink, *D* is the diffusion coefficient of the molecules in the ink, *v*_0_ is the molecular volume, and σ is the surface energy of the films. Here, we define two dimensionless time constants: time delay for nucleation (τ_d_ = *T*_d_/*T*_e_) and time available for solvent evaporation after critical supersaturation [τ_a_ = (*T*_e_ − t^*^)/*T*_e_]. Nucleation and aligning can only occur if the nucleation process is completed before the complete solvent evaporation (τ_a_ > τ_d_) (Fig. 2A (i), and red curve in Fig. 2B). If τ_a_ < τ_d_, the solvent completely evaporates before the molecules can nucleate or align on the fly, resulting in the formation of random aggregates [Fig. 2A (ii)]. If τ_a_ > > τ_d_, the deposited wet nanodroplets can rapidly aggregate into a thin liquid film, and nucleation will be influenced by solid-liquid interfaces [Fig. 2A (iii)]. In summary, aerosols in-flight self-assembly can be achieved under the following conditions

$$\begin{array}{c} \tau_{d}<\tau_{a},\frac{32}{\pi^{2}}\frac{v_{0}\sigma^{2}}{\gamma^{*}{\left({\mathrm{kt}}_{d}\right)}^{2}{\mathrm{DNc}}_{e}\left(S+1\right){\text{ln}}^{3}\left(S+1\right)}\frac{8D_{v}{\mathrm{MP}}_{d}}{R\rho_{d}d_{i}^{2}t_{d}}\\ <1-\frac{t^{*}8D_{v}{\mathrm{MP}}_{d}}{R\rho_{d}d_{i}^{2}t_{d}} \end{array}$$ (3)

To demonstrate the role of electrohydrodynamic aerosolizing and in situ electrical poling, we examine the crystallization results under different conditions. For the glycine/PVP films synthesized via direct solidifying bulk solution under a high electric field (100 kV/mm), only α-glycine crystals are detected (black curve in Fig. 2B). A high-temperature TEA printing process (>60°C) causes α-polymorphs glycine films (dark red curve in Fig. 2B and fig. S8), which is inconsistent with our above statement (forming amorphous glycine because of τ_a_ < τ_d_). One possible explanation is that if the crystallization goes through the amorphous state, the obtained amorphous glycine is of low physical stability and will spontaneously transform into a more stable crystalline state (α-glycine). Electrohydrodynamic-focused printing causes microdroplets, and microglycine crystals (fig. S9). Without nanoconfinement, the mixture of α- and β-polymorphs is obtained (purple curve in Fig. 2B). In this case, aligning the polarization of microcrystals under a limited electric field strength is extremely challenging, and the resulting β-glycine crystallites are preferentially orientated with their *ab* crystallographic planes parallel to the films. In the absence of a thermal field promoting supersaturation, most nucleation occurs heterogeneously based on interfacial effect (τ_a_ > > τ_d_). The β-glycine crystals with a dominant orientation [001] are obtained (blue curve in Fig. 2B). For high-viscosity glycine/PVP inks, electrospun nanofibers tend to appear instead of nanodroplets (*40*). In this process, the β-polymorph is formed because of the confinement of the nanofibers, and the β-glycine nanocrystals tend to grow along their native fast-growth direction parallel to the one-dimensional pore direction, giving rise to the [001] peak (fig. S10).

Different electrohydrodynamic jetting modes are formed depending on process parameters (fig. S11). Stable jetting modes can form individual nanodroplets continuously, which is favorable for the TEA printing process (fig. S12 and note S3). To understand the electrohydrodynamic jetting behavior, we systematically investigate the microfluidic formulation and printing optimization by experimental process (using high-speed camera imaging). Here, we define the dimensionless flow rate (μ) and dimensionless voltage (ν) to regulate the electrohydrodynamic jetting process. Depending on the relationship between applied flow rate (*Q*) and critical flow rate (Q^*^)

$$\mu =Q/\left(10\cdot Q^{*}\right)=Q/\left(10\cdot {\gamma \varepsilon }_{0}\varepsilon /\mathrm{\rho K}\right)=\left(\rho \mathrm{KQ}\right)/\left(10{\gamma \varepsilon }_{0}\varepsilon \right)$$ (4)

where ε_0_ is the permittivity of free space, and *K*, γ, ε, and ρ are the conductivity, surface tension, permittivity, and density of fluid, respectively. Dimensionless voltage (ν) is defined as the ratio of applied voltage (*V*) and the critical voltage (V^*^)

$$\upsilon =V/100\cdot V^{*}=V/100\cdot \sqrt{\gamma d/\varepsilon_{0}}=\left(V\sqrt{\varepsilon_{0}}\right)/\left(100\cdot \sqrt{\gamma d}\right)$$ (5)

We explore the jetting map (gray domain in Fig. 2C) based on various jetting modes in terms of two dimensionless parameters. The points where the transition of stable jetting mode occurs are identified by performing the experiments with various μ values (from 0.1 to 10) and the corresponding ν values. Then, two curves are fitted to these points, which give the boundary of the stable jetting mode domain. We also measure the grain size of the films and find that the crystals are [001]-oriented β-polymorphs when the grain size is >1 μm and [020]-oriented β-polymorph when the size decreases to <0.5 μm (Fig. 2C and fig. S8, S13, and S14). These results indicate that the grain size should be <0.8 μm (orange domain in Fig. 2C) for homogeneous nucleation and electrical poling. The depositing rate is controlled by the ink flow rate during the stable jetting mode, where the thickness of the film increases with the ink flow rate monotonically (fig. S15). Other parameters are systematically investigated to optimize the printing process (fig. S16 and tables S3 and S4).

### Mechanical properties and thermostability

**Fig. 3.**
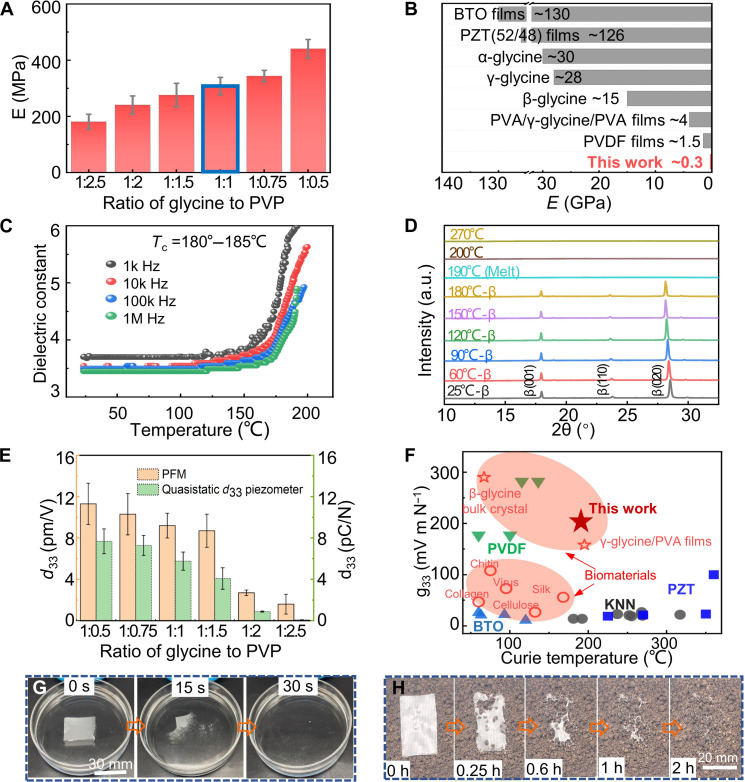
Mechanical and piezoelectric properties, thermostability, and degradability of glycine/PVP films. (**A**) Elastic moduli of the glycine/PVP films fabricated with different glycine-to-PVP mixing ratios. E, elastic modulus. (**B**) Elastic moduli of the as-deposited glycine/PVP films compared with the piezoelectric ceramics (PZT and BTO), piezoelectric polymer (PVDF), and glycines. (**C**) Dielectric constant as a function of temperature for as-deposited glycine/PVP (1:1) films. The dielectric constants are measured at 1 kHz, 10 kHz, 100 kHz, and 1 MHz. (**D**) In situ variable temperature XRD spectra of as-deposited glycine/PVP (1:1) films. (**E**) Piezoelectric coefficients, *d*_33_ of glycine/PVP films with different composition ratios. (**F**) Comparison of longitudinal piezoelectric voltage coefficient *g*_33_ of most actively studied piezoelectric materials with our glycine/PVP (1:1) films as a function of Curie temperature *T*_C_. (**G**) The solubility test of the glycine/PVP (1:1) films in deionized water. (**H**) The biodegradability test of the glycine/PVP (1:1) films under moist soil.

The intrinsic rigidity of glycine crystals poses a challenge for the application of flexible devices. The elastic behaviors of films are examined under tensile stimuli. Films with higher polymer content (glycine-to-PVP ratio < 1:1) show substantially enhanced stretchability, with tensile strains greater than 0.3% (fig. S17). With the increase of mixing ratio of glycine to polymer, films show a monotonic increase of Young’s modulus (from 180 to 440 MPa; Fig. 3A). Their Young’s moduli are nearly two orders of magnitude smaller than glycine crystals and three orders of magnitude smaller than PZT (Fig. 3B and tables S5 and S6). Materials with such modulus may cover a range of tissues (fig. S18) and are suitable in cartilage/bone tissue engineering. The flexibility of the films remained consistently low under a repeating 0.01 to 0.2% strain in the frequency sweep mode from 0.1 to 80 Hz (fig. S19, A and B). This range covers the frequency range of most biomechanical movements (0.5 to 5 Hz). Their moduli-temperature dependence is shown in fig. S19 (C and D). In addition to the large ratio of area to thickness, the flexibility of the films is resulted from the composite nanocrystalline-polymer structure (note S4). The soft and continuous PVP could effectively dissipate the mechanical impacts on the fragile glycine crystals. Meanwhile, the nanoscale glycine crystals seamlessly encapsulated by PVP also largely minimize the defective spots of the film and are favorable for their flexibility.

Curie temperature is a thermo-characteristic point, above which ferroelectrics will lose their persistent piezoelectricity. Here, we investigate the thermostability of the as-deposited β-glycine film. Figure 3C shows the dielectric constant (ε*_r_*) versus temperature for the glycine/PVP films. The measured dielectric constant at different frequencies shows no evident anomaly before ~180°C while a sharp change in the range of 180 to 190°C (Fig. 3C and fig. S20). Variable-temperature x-ray diffraction (XRD), differential scanning calorimetry (DSC), and thermogravimetric analysis (TGA) reveal the disappearance of β-glycine diffraction peaks at ~185°C, and only β phase is exhibited before the melting temperature (Fig. 3D and fig. S21). These results indicate that the sharp change of the dielectric constants is due to the melting of β-glycine nanocrystalline films. The melting temperature is below the glycine decomposition temperature (252°C, fig. S21), suggesting that the disappearance of β-glycine is due to melting without decomposition. The piezoelectric failure temperature *T*_c_ of the β-glycine/PVP films is between 180° and 185°C. β-Glycine nanocrystals fabricated by TEA printing exhibit increased stability against phase transitions at elevated temperatures (Fig. 3D and fig. S21) compared with the bulk form, which is known to be metastable and will transform to α form at 67°C (*41*). The XRD peaks vanish when the films are heated above 250°C because of decomposition. TGA results reveal one mass loss process, with an apparent onset at ~200°C and an increased rate of mass loss as the temperature approaches ~255°C. Metastable β-glycine becomes stable when its crystal size is confined to nanoscale dimensions. The size confinement effect reduces the melting point of β-glycine below the temperature where melting occurs concomitant with decomposition, which can be explained through the Gibbs-Thomson relationship (note S5).

### Piezoelectric properties and degradability

The piezoelectric coefficient *d*_33_ of the glycine/PVP films is measured by piezoresponse force microscopy (PFM) and a *d*_33_ meter (fig. S22). The *d*_33_ of the films with a glycine-to-PVP ratio of 1:1 is 8 to 10.4 pm/V (Fig. 3E), which is superior to most reported natural biomaterials (fig. S23 and tables S5 and S7). The PFM mapping of the out-of-plane (OOP) amplitudes of the glycine/PVP films showed a uniform piezoelectric response (fig. S24A). The uniform OOP phase mapping and phase histogram further confirm their well-aligned polarization (fig. S24, B to D). The OOP amplitude increases linearly with increasing the AC voltage, and the effective piezoelectric coefficient is around 10.8 pm V^−1^ (fig. S24E). A slightly decreasing trend of *d*_33_ with the increase of films thickness is observed (fig. S24F). Extensive depositing time would cause more undesired structures defects on the films, which is not favorable for their piezoelectric performance. To further demonstrate the macroscopic piezoelectricity of the glycine films, we fabricate a cantilever vibration system based on glycine/PVP films (fig. S25). The cantilever test is effective in eliminating errors stemming from variations in parasitic capacitance or triboelectricity. It ensures that the voltage output is genuinely attributed to the glycine/PVP films. The relatively high piezoelectric coefficient is attributed to the excellent polarization alignment by electrical poling, which let most of the (020) polar surface orient toward the OOP direction. We compare the *g*_33_ of our glycine/PVP films with some actively studied piezoelectric materials as a function of Curie temperature (Fig. 3F and tables S5 and S8). Our glycine/PVP films exhibit a high *g*_33_ (~190 × 10^−3^ Vm N^−1^), which is superior to that of most piezoelectric crystals, ceramics, and natural biomaterials. In addition, the piezoelectric failure temperature of our glycine/PVP films is comparable to the Curie temperature of commercial PZT-5H and much higher than that of polymer PVDF and most natural piezoelectric biomaterials.

Because both PVP and glycine are water soluble, the glycine/PVP films can be dissolved into an aqueous solution within 30 s (Fig. 3G and movie S2). The glycine/PVP films also exhibit excellent biodegradability in the natural environment. The glycine/PVP sheet undergoes a swelling process and then becomes fractured after 0.25 hours at the soil’s surface. Eventually, it becomes completely biodegraded after 2 hours (Fig. 3H and movie S3).

### Transcutaneous ultrasound energy harvesting

**Fig. 4.**
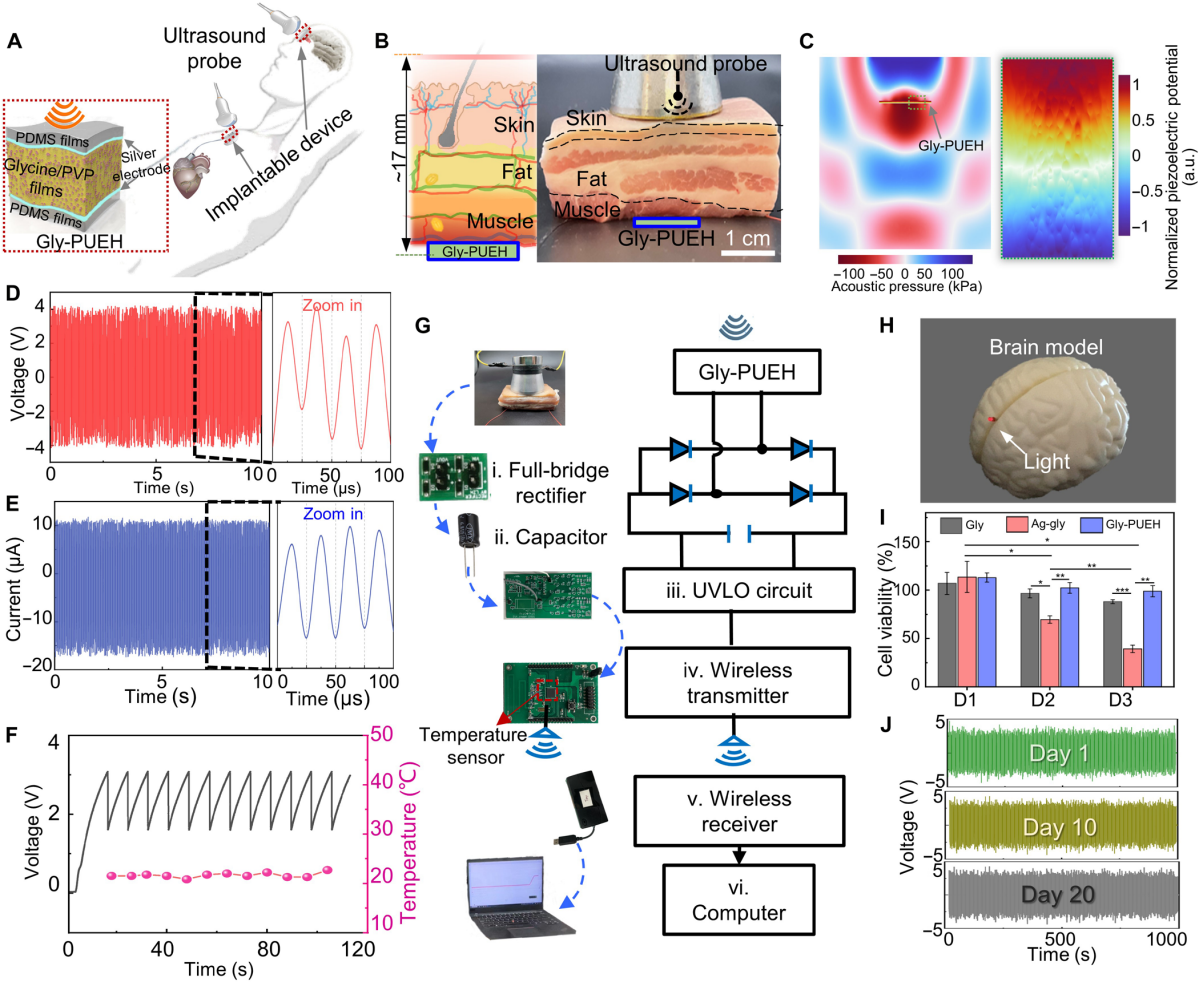
Gly-PUEH characterization ex vivo porcine tissue. (**A**) Scheme of through-tissue wireless ultrasonic energy transfer by implanted Gly-PUEH device. (**B**) Schematic and picture of the Gly-PUEH implanted at ~1.7 cm under the porcine tissue. (**C**) Finite element simulation of the ultrasonic-induced piezoelectric potential distribution in the Gly-PUEH. (**D** and **E**) Open-circuit voltage and short-circuit current signals of the Gly-PUEH. (**F**) Charging curve of 33-μF capacitor and the corresponding transmitted signals. (**G**) The wireless communication configuration with a transmitter supplied by the Gly-PUEH device ex vivo. (**H**) Optical image showing one LED lit up by the Gly-PUEH for optical stimulation in a brain tissue phantom. (**I**) Cell viability characterization of myofibroblast cells cultured with sample extracts using the CCK-8 method (Gly, glycine films; Ag-gly, Ag-coated glycine films). (**J**) Time-dependent piezoelectric voltage outputs of the Gly-PUEH.

Piezoelectric biomaterials are intrinsically suitable to couple mechanical and electrical energy in biological systems for in vivo electricity generation. However, the electricity generated solely from random and low-frequency biomechanical movements is insufficient to power most currently implantable medical devices, in particular when taking into account the relatively low power output of piezoelectric biomaterials. To address this limitation, external ultrasound devices can provide safe and on-demand charging for implantable nanogenerators through wireless energy transfer using ultrasound waves. As a proof of concept, we demonstrate a glycine-based PUEH (Gly-PUEH) as an external charging system for electronic transcutaneous implants (Fig. 4A). The Gly-PUEH has a thickness of ~2 mm and exhibits excellent flexibility (fig. S26). The ex vivo tests are performed by inserting it under ~17-mm porcine tissue (Fig. 4B). The simulations predict the sound field distribution within tissues and the normalized electric output of piezoelectric biofilm (Fig. 4C, figs. S27 to S29, table S9, movies S4 and S5, and note S6). The Gly-PUEH generates a voltage of ~3.6 V, current of ~10 μA, and power density of ~35 μW cm^−1^ (Fig. 4, D and E, and fig. S26F), high enough to recharge some implants (e.g., pacemaker and cardioverter-defibrillator). Its high-power output is due to the remarkable electromechanical properties of glycine films. “Soft, flexible” characteristics enable the placement of Gly-PUEH onto various target locations of the body, and the “transient” characteristics eliminate the need for surgical removal. The control experimental results further confirm that the electrical signals are generated by the glycine/PVP films (fig. S30). We further demonstrate the Gly-PUEH for powering a temperature monitoring sensor ex vivo (Fig. 4F and fig. S31). The energy converted from the Gly-PUEH is stored in a capacitor (33 μF), followed by an undervoltage lockout (UVLO) module that guarantees the activation of the wireless transmitter with enough energy. The transmitted data are then collected by a wireless receiver every 8 s and shown on the computer screen through the USB serial port (Fig. 4G and movie S6).

Neuroscience research using optogenetics has created important avenues for the understanding of neural circuitry and the treatments of neurological diseases. Continued advances are hampered, however, in part due to the technological limitations in physical tethering of connection of external devices with probes inserted into delicate regions of the brain. Ideally, this limitation can be overcome by using a wireless, battery-free implanted microsystem that combines a degradable flexible power supply with light-emitting diode (LED) probes. The demonstration experiments using red LED and brain model illustrate this operation. Following a wirelessly transcutaneous ultrasound triggering, the LED will be initiated for optogenetic stimulation or inhibition (Fig. 4H). To specifically investigate the biocompatibility of Gly-PUEH, we culture myofibroblasts WPMY-1 with glycine films, Ag-coated glycine films, and Gly-PUEH extraction medium and evaluate the cell viability using the cell counting kit-8 (CCK-8) assay at the following day 1, day 2, and day 3 (Fig. 4I and fig. S32). On day 1, the three groups keep comparable cell viability. However, the cell viability of the Ag-coated glycine films shows decreasing in the following days, demonstrating that the Ag coatings produce a high cytotoxicity. For the Gly-PUEH samples, the myofibroblasts exhibit a high proliferation rate and the viabilities are still comparable to the control group (fig. S32) on day 3, indicating the Gly-PUEH does not exhibit cytotoxicity and has good biocompatibility. The voltage output of the Gly-PUEH remains nearly unchanged in 20 days (Fig. 4J), indicating its excellent durability.

### Outlook

The TEA printing method enables one-step high-speed fabrication of piezoelectric bio-organic films using rapid aerosol-based assembling, poling, and depositing. This strategy can be generalized for different piezoelectric biomaterial systems, greatly expanding the piezoelectric biofilms library for emerging applications. The next phase of research will focus on leveraging the TEA printing and piezoelectric biomaterial libraries, as well as machine learning–guided design strategies to accelerate the development of a broad range of piezoelectric biomaterials for flexible bioelectronics and biotissues therapeutics. Although the glycine films fabricated by the TEA printing method exhibit preferable electromechanical properties, there are still several challenges in integrating these films into biomedical applications. The underlying piezoelectric mechanisms of the biomaterials are still not clearly understood, and piezoelectric performance remains low compared to inorganic ceramics and organic polymers. Disalignment of maximum piezoelectric constant modes with the applied direction is rather challenging. The high Young’s modulus and poor elastic recovery make the studied biofilms incompatible with the particular soft bioelectronics. Given the piezoelectric implants for electrical stimulation, the safe range of electricity produced by the biomaterials compatible with the tissue functions remains unclear.

## MATERIALS AND METHODS

### Glycine/PVP composite ink formulation

PVP in water solution (10 to 20% w/v) is prepared by dissolving PVP powders (average Mw, 1,300,000) in deionized water, and is stirring for 5 hours at 90°C. Glycine solution (20% w/v) is obtained by dissolving 20-g glycine powders (Macklin/AR9, 9.5 to 100.5%) in 100-ml deionized water. Ten grams of 20% glycine solution is added into 10 g of 15% PVP solution to obtain a glycine/PVP composite ink with glycine-to-PVP ratio of 1:1. With the same manner, other different glycine-to-PVP ratios, 1:2.5, 1:2, 1:1.5, 1:0.75, and 1:0.5, of the inks are obtained. A homogeneous composite ink is obtained by constantly stirring the mixture for 5 hours using a magnetic rotator. PVP is chosen due to its biocompatibility, nonantigenicity, and easy processability.

### TEA printing

The components of the TEA printer can be found in table S1. A control panel controls the R2R coating system. Real-time substrate moving velocity and roller temperature are tracked during the aerosol printing. The ink flow rate is actively controlled with respect to ink properties to achieve uniform films. A home-made printing nozzle panel with nine nozzles is applied to disperse the aerosolized ink for high-throughput printing. During the TEA printing process, a heating stage (60°C) is used to quickly evaporate the solvents of deposited wet films, and a heating light (100°C) is used to accelerate the nanodroplets to saturation state. The size of the aerosolized droplets depends on some process parameters (table S4). In comparison with the fabrication process of piezoelectric ceramics, which typically requires high-temperature sintering of ≥1200°C followed by high-voltage polarization, TEA method can synthesize piezoelectric bio-organic films at temperature ≤ 60°C in one-step fabricating process.

### Fabrication of Gly-PUEH

The Gly-PUEH is designed on the basis of a ~200-μm-thick glycine/PVP (1:1) film (4 × 4 cm^2^). The thin ~3.5 cm by 3.5 cm Ag electrode is coated on both film surfaces. We seal the film and electrode using polydimethylsiloxane (PDMS; Sylgard 184, Dow Corning Co. Ltd). Ag thin films with a thickness of ~10 μm are deposited on both sides of the flexible glycine/PVP film (4 × 4 cm^2^, thickness of ~200 μm) as electrodes. Then, the Ag-deposited film is packaged by PDMS films. The uncured PDMS with 10 wt % curing agent is used to spin-coating encapsulation layers.

### Characterizations

#### Structural characterizations

The morphologies of films are observed by an SEM (FEI Quanta 450). The XRD patterns of glycine/PVP films are measured by a wide-angle x-ray diffractometer (PANalytical X’pert3 diffractometer) using Cu Kα radiation of wavelength 1.54060 Å. The XRD data are obtained in the range of 10° to 50° (2θ) using a step size of 0.02° at room temperature. Chemical compositions of the films are measured by FTIR spectroscopy (Bruker Tensor 27 FTIR).

#### Mechanical characterizations

The stress-strain relationships and tensile moduli of the glycine/PVP films are measured by tensile test using tensile tester system (TY8000-A, Tian Yuan Test Instrument) according to ASTM D882. All specimens with dimension of 50 mm × 10 mm × 0.1 mm are tested at 25°C and 50% relative humidity, and the average value of five test trials is taken for each specimen. The dynamic modulus of glycine/PVP films are measured by a dynamic mechanical analyzer (TA Q800).

#### Thermostability measurements

To characterize the temperature-induced phase transition, we perform the in situ variable temperature XRD, DSC, and TGA experiments on the as-deposited films. XRD measurements are performed using a Rigaku Smartlab with Cu Kα radiation of wavelength 1.54060 Å. The sample is heated continuously at a rate of 10°C min^−1^ up to a maximum of 270°C. During this, data are collected with a scanning speed of 5° min^−1^. DSC measurements are performed using a DSC 3 (METTLER TOLEDO). TGA is performed with a TGA/DSC 3+ (METTLER TOLEDO).

#### Electromechanical properties measurements

The dielectric constant (ε_33_/ε_0_) and dielectric loss (tanδ) are measured as a function of temperature (25° to 200°C) using an LCR meter (Agilent E4980A, Santa Clara, CA) under a small alternating voltage of 1 V. In the dielectric measurement, the temperature is controlled at a heating rate of 3°C/min using a programmable temperature controller. The *d*_33_ values are determined by a quasi-static piezoelectric meter (YE2730A *d*_33_ meter), and the average value of five test trials is taken for each specimen. PFM characterizations are conducted on an Asylum Cypher ES AFM system, applied to a Cr/PtIr-coated conductive probe (Nanoworld Arrow-EFM). The effective piezoelectric coefficient *d*_33_ is measured with the AC voltages of 4 V.

#### *Calculation of piezoelectric voltage coefficients (g*_33_)

The piezoelectric voltage coefficients (*g*_33_, a key factor for piezoelectric receiving transducers) are calculated from experimentally determined *d*_33_ and film’s permittivity ε_r_: 𝑔_33_ = 𝑑_33_/(ε_0_ × ε_r_).

#### Solubility and biodegradability tests

To evaluate the solubility, the glycine/PVP films (30 mm × 30 mm × 15 μm) are placed at the surface of deionized water. To evaluate the biodegradability, the glycine/PVP films (20 mm × 40 mm × 50 μm) are placed at the surface of natural soil (relative soil moisture of 60%). The samples are monitored periodically to assess the degree of biodegradation.

#### Ultrasonic-induced outputs of the Gly-PUEH

We characterize the Gly-PUEH under porcine tissue, which closely resembles human skin in terms of composition and anatomy. The Gly-PUEH device is placed under porcine tissue (thickness of ~17 mm). An ultrasonic transmitter is driven by an ultrasonic generator (K3, KMD) with a constant input frequency of 40 kHz and input power of 0.72 W cm^−2^. The input power intensity is measured by a hydrophone (J100E, CYS).

### Wireless signal transmission

A wireless temperature monitoring system ex vivo is powered by the Gly-PUEH. For the wireless temperature monitoring system, the transmitter sends a real-time radio frequency signal obtained by a temperature sensor integrated with the transmitter. The working process is described as follows. A full-bridge rectifier is first connected to the Gly-PUEH device for rectifying the AC to the DC. Then, the converted energy is stored in a 33-μF capacitor. To guarantee that the wireless MCU with the working voltage of ~3 V can be activated with enough energy gathered in the capacitor to complete a successful signal transmission, we insert an UVLO module between the full-bridge rectifier and the wireless transmitter. The rising switching-on and falling switching-off voltages is set to 3 and 1.6 V, respectively. After completing the signal transmission, the corresponding wireless receiver reads out the data, sends it to the computer, and displays it through the USB serial port. Regarding the wireless transmission clarification, while Bluetooth (2.4 GHz) is a commonly used wireless protocol, we used a 433-MHz wireless transmission protocol in our demo. This lower frequency is advantageous for wireless transmission through thicker biological tissue due to its better penetration capabilities. In the experiment, we use the integrated temperature sensor to detect the room temperature variation every 8 s. In our demonstration, the wireless signal transmission system looks bulky because it is not highly integrated. The demonstration is only proof to showcase the feasibility of Gly-PUEH powering a wireless sensor beneath biological tissue. For practical implant applications, future work can be done to integrate the separate module into a compact wireless sensor by using small-footprint components and an optimized layout.

### In vitro biocompatibility

Myofibroblast cell line WPMY-1 is purchased from American Type Culture Collection and is cultured in Dulbecco’s modified Eagle’s medium (Gibco, catalog no. 11875085, USA) supplemented with 5% fetal bovine serum (Gibco, catalog no. 10270106, USA), and 1% penicillin-streptomycin (Gibco, catalog no. 15140122, USA). Cells are passaged at 80% confluence and cultured under 5% CO_2_ humidified atmosphere at 37°C.

Glycine films, Ag-coated glycine films, and Gly-PUEH are exposed under ultraviolet light for sterilization. The sterilized samples are immersed into the completed culture medium for 24 hours at 37°C to obtain the extraction medium, following the instruction of GB/T 16886.12/ISO 10993-12. The cells are seeded into the 96-well plates at a density of 2.5 × 10^3^ ml^−1^ with 0.1-ml completed culture media. The completed culture medium is disposed of after 24-hour incubation, and the extraction medium is used for the evaluation. Cell viability is evaluated using the CCK-8 assay on the following day 1, day 2, and day 3, wherein the absorbance of the treated supernatant was measured at 450 nm. Meanwhile, the cells are stained with 5 μM calcein AM (Invitrogen, catalog no. C3100MP, USA) under 37°C for 20 min and 5 μM propidium iodide (Sigma-Aldrich, catalog no. 81845, USA) at room temperature for 1 min to identify the live and dead cells at each time point. The stained samples are imaged with a confocal microscope (Leica, SP8, Germany). All fluorescent images are processed by ImageJ software (National Institutes of Health, USA).

The results are expressed as means ± SD of three independent trials. Student’s *t* tests are conducted to evaluate associations between independent variables, and the statistical significance is defined as *P < 0.01, **P < 0.001, and ***P < 0.0001.

## Supplementary Materials

### The PDF file includes:

Supplementary Notes Figs. S1 to S32 Tables S1 to S9 Legends for movies S1 to S6 References
