# Supplementary material for: One-step high-speed thermal-electric aerosol printing of piezoelectric bio-organic films for wirelessly powering bioelectronics
Source: Sci Adv. 2024 Oct 25;10(43):eadq3195. doi: 10.1126/sciadv.adq3195 (PMC11506135; doi:10.1126/sciadv.adq3195)
Supplement: Supplementary file 1 — Supplementary Notes Figs. S1 to S32 Tables S1 to S9 Legends for movies S1 to S6 References [file sciadv.adq3195_sm.pdf]

Science Advances
AAAS

Supplementary Materials for

One-step high-speed thermal-electric aerosol printing of piezoelectric bio-organic films for wirelessly powering bioelectronics

Xuemu Li *et al.*

Corresponding author: Zhengbao Yang, zbyang@ust.hk

Sci. Adv. **10**, eadq3195 (2024)
DOI: 10.1126/sciadv.adq3195

The PDF file includes:

Supplementary Notes
Figs. S1 to S32
Tables S1 to S9
Legends for movies S1 to S6
References

Other Supplementary Material for this manuscript includes the following:

Movies S1 to S6

Supplementary Notes

Note S1. Piezoelectricity of glycine and nanoconfinement effect

Amino acids, as the basic building blocks of proteins, exhibit structure-dependent piezoelectric properties, and are abundant in nature. Glycine is the simplest amino acid and stands out with its strong piezoelectric property.

Glycine is the only non-chiral amino acid, and it can crystallize into three polymorphic forms, α, β, and γ-glycine. α-glycine excludes piezoelectricity due to its central symmetry point group. β-glycine and γ-glycine are noncentral symmetry point group, which induce piezoelectric effect. The most noteworthy of the recent studies is that the piezoelectric coefficient d_{16} of β-glycine reaches about 200 pm V^{-1} (*42*). This value of piezoelectric coefficients is comparable to or higher than that of the conventional piezoelectric inorganic or organic materials. β and γ-glycine crystals have also been proved to be ferroelectricity, so their polarization directions can be controlled by an external electric field (*43*, *44*). However, it is extremely difficult to align the internal dipoles of the crystallites (domains) within the biological systems because of their complex and heterogeneous structures. For example, glycine requires an electric field exceeding GV/m for domain aligning, which is 3-4 orders of magnitude higher than PZT. In addition, the crystal class of β-glycine is the most difficult to form in kinetics and the most unstable in thermodynamics under ambient conditions, as the metastable polymorph can spontaneously transform into stable α- or γ-phases under ambient conditions. For example, the bulk β-glycine crystals are the least stable and readily transform to α-glycine in moist air after being left at room temperature for several hours or heated to 67 °C.

With the increasing surface-area-to-volume ratio due to the reduced crystal sizes, the nanometer-scale crystals always exhibit unique properties (*45*). According to the classical nucleation theory, small clusters of molecules form in the early stage, and then grow into a nucleus. The total free energy of the nucleus can be expressed as the combined contribution of the volume free energy change ΔG_V and the opposing surface free energy ΔG_S. The volume free energy is always negative and stable, which can be attributed to the intermolecular bonding energy, while the surface free energy is positive and unstable, which corresponds to the interface formation between the nucleus and surroundings (*46*). When considering a spherical nucleus, the free energy during the process of crystallization can be characterized in the following manner:

$$\Delta G_{cryst} = \Delta G_V + \Delta G_S = \frac{4}{3}\pi r^3 \Delta g + 4\pi r^2 \sigma \qquad \text{(S1)}$$

where r represents the radius of the spherical nucleus, Δg denotes the energy difference between the nucleated phase and the nucleating phase per unit volume, and σ represents the surface tension at the interface, which represents the surface energy per unit area. Equation (S1) clearly shows the strong relationship between ΔG_{cryst} and the size of the crystal. By taking the derivative of ΔG_{cryst} with respect to r, the maximum value of ΔG_{cryst} can be obtained, which corresponds to the activation energy of nucleation, ΔG_c, at the critical radius, r_c. Overcoming this energy barrier is crucial for spontaneous nucleation. Dissolution occurs when the nucleus is smaller than r_c, whereas crystallization occurs when the nucleus is larger than r_c. The critical size of organic nuclei typically falls within the range of a few nanometers to tens of nanometers (*47*).

The surface energies, volume free energies, and crystal morphologies of different polymorphs vary due to their unique crystal structures. Consequently, each polymorph is likely to exhibit distinct values of ΔG_{crys} and r_c, where the realms of thermodynamics and kinetics intersect. At the critical

size, the discrepancy in kinetic barriers between the two polymorphs corresponds to the disparity in their thermodynamic stability. Consequently, when examining the nucleation pathway of different forms, it is anticipated that they will possess different critical sizes and corresponding nucleation barriers (*45*). In Figure S1b, the free energy profiles illustrate that the α phase glycine crystals exhibit greater stability in larger bulk sizes, while the metastable β phase glycine crystals are more stable at the critical size and slightly beyond. When the dimensions slightly exceed the critical size, the β phase becomes the thermodynamically favored phase, characterized by the lowest free energy and a lower kinetic barrier compared to other phases. This ranking can persist beyond the critical size, but as the size continues to increase, it reverses to the stability ranking observed in bulk materials. This observation highlights that the β-glycine, which is typically considered a metastable form, actually becomes the stable form when the crystal size is confined to nanometer-scale dimensions.

Note S2. The size of electrosprayed droplets

The jet disintegrates downstream and generates charged droplets, forming a spray. The Taylor-cone jet mode can generate monodisperse droplets because the axisymmetric surface wave instability dominates the jet break-up, leading to a constant droplet-to-jet diameter ratio.

Since the droplets are charged, columbic repulsion disperses them in the form of a spray. The charged droplet travels in the electric field, and solvent from the droplet evaporates. As the solvent evaporates, the droplet shrinks, while the charge on the droplet remains constant, causing the increase of the surface charge density. When the electrostatic repulsion force between charges overcomes the surface tension force, the value will increases to a limit, known as Rayleigh limit. At the Rayleigh limit, droplet fission occurs and smaller droplets are generated. The size of droplet at Rayleigh limit where the droplet with a surface energy per unit area σ, and a charge q is:

$$\mathrm{r}_{rayleigh} = \left(q^2 \big/ 64\pi^2 \varepsilon\sigma \right)^{1/3} \qquad \text{(S2)}$$

The size of the electrosprayed droplets can be depend on the properties of inks (such as surface tension (γ), electrical conductivity (K), viscosity (μ), relative permittivity (ε_r) and density (ρ)) and the process parameters (such as flow rate (Q), applied voltage (V), and substrate-to-capillary distance (d)). These parameters can be combined into three dimensionless parameters: relative permittivity ε_r, dimensionless flow rate $\eta = (\frac{Q}{\gamma\varepsilon_r\varepsilon_0 / \rho K})^{1/2}$, and viscosity number $\Pi_\mu = \frac{1}{\mu}(\frac{\gamma^2\rho\varepsilon\varepsilon_0}{K})^{1/3}$.

Some previous studies confirmed a negligible effect of viscosity on the droplet size, in a very broad range of $\Pi_\mu > 0.022$, encompassing most liquids (*48*). Also the droplet size is independent of the capillary size as long as the electrospray is kept in cone-jet mode.

The droplet size can be calculated based on the semi-empirical formulations:

$$D_\mathrm{d} = 3.78\pi^{-2/3} 0.6 Q^{1/2} \left(\frac{\rho\varepsilon_0}{\gamma K} \right); \varepsilon_r < 100, \eta < 2, K < 10^{-7}\, S/cm \qquad \text{(S3)}$$

$$D_\mathrm{d} = G(\varepsilon_r)\left(\frac{Q\varepsilon_r\varepsilon_0}{K} \right)^{1/3}; G(\varepsilon_r) = 10.87\varepsilon_r^{-6/5} + 4.08\varepsilon_r^{-1/3}; \varepsilon_r < 100, \eta < 2, K > 10^{-7}\, S/cm \qquad \text{(S4)}$$

$$D_d \sim \left(\frac{\rho Q^2}{2\pi^2\gamma}\right)^{1/3};\varepsilon_r < 100, \eta > 2, K > 10^{-7} S/cm \qquad \text{(S5)}$$

$$D_d = 6.64\varepsilon_r^{-1/6}\left(\frac{\rho\varepsilon_r\varepsilon_0}{K}\right)^{1/3};\varepsilon_r > 100, \eta < 2, K > 10^{-7} S/cm \qquad \text{(S6)}$$

For solutions with high conductivity, charge transport through jet is rapid, hence droplet diameter is independent of electrostatic parameters. Whereas at low conductivity, it decreases with the increase of the applied voltage (Eq. (S3)). Based on above formulations and the physical properties of inks (Table S1), the calculated droplet size is ~0.69 μm.

Note S3. Electrohydrodynamic jetting and important factors

During the electrohydrodynamic jetting, the micro fluid experiences surface charge convection and conduction (Figure S12A). The charge convection toward surface causes the electrical normal stress. The applied flow rate (Q) and electrical charging time, which is related with the capacitance (ε) and resistance ($1/K$) of the system, are the variables affecting the charge convection. The surface charges conduction lead to the electrical tangential stress along the surface meniscus toward the cone apex. The charge acceleration speeds up the surrounding fluid sequentially, then a jet is formed by these charge and fluid motions. The variables influencing this charge conduction are the electric field (E), the charge amount represented by the permittivity of fluid (ε), and flow resistance represented by the viscosity of fluid (η). The hydrodynamic force (F_h) can supply fluid to the meniscus, and the surface tension force (F_γ) will hang the droplets on the capillary tip.

The formation process of an electrohydrodynamic jet can be calculated using a set of equations in which electrical and mechanical forces are included (*49*),

$$\frac{\partial\rho_i}{\partial t} + \nabla\cdot(\rho_i v_i) = \sum_{j=1}^{n} I_{ij}, \qquad \text{(S7)}$$

$$\frac{\partial\rho_i v_i}{\partial t} + \nabla\cdot(\rho_i v_i \otimes v_i) = \nabla\cdot\prod_{\ell} + \rho_i g + \sum_{j=1}^{n} P_{ij} + L_\ell \qquad \text{(S8)}$$

where i refers to the gas or liquid phase. The symbol $\otimes$ is the dyadic product of the vectors. The stress tensor on the liquid surface is as follows,

$$\prod_{\ell} = Grad(lp) + \eta_\ell \nabla v_\ell \qquad \text{(S9)}$$

where p is the dynamic pressure and η is the liquid viscosity. *Grad* is the different operator on a scalar. This difference is due to the pressures on both sides of the interfacial surface ($Grad(p) = p_\ell - p_g$). Then the force density on the liquid jet is obtained depending on Eq. (9),

$$\frac{\partial\rho_\ell}{\partial \mathrm{t}} = \rho_\ell g + L_\ell - \phi_{st} - \nabla\cdot(Grad(lp)) + \eta_\ell\nabla v_\ell + \rho_\ell v_\ell \otimes v_\ell \qquad \text{(S10)}$$

Note S4. Flexibility of glycine/PVP films

The flexibility of glycine/PVP films was primarily resulted from the very large ratio of area to thickness. The flexibility of a material can be represented with the flexural stiffness *D*:

$$D = \frac{Et^3}{12(1-v^2)} \qquad \text{(S11)}$$

where E is the elastic modulus, t is the thickness of the films, and v is the Poisson's ratio. The flexural stiffness is proportional to the cube of the thickness, so the films with ultrathin thickness can exhibit excellent flexibility. The critical bending radius r_c of the films can be estimated as:

$$r_c = \frac{t}{2\varepsilon_{failure}} \qquad \text{(S12)}$$

where t is the thickness of the films, and $\varepsilon_{failure}$ is the fracture strain. For the pure glycine crystal films ($\varepsilon_{failure}$ is ~0.93‰) with a thickness of 30 µm, the theoretical bending radius limit is 32 mm. Our as-deposited films can easily experience larger deformation with a bending radius of the films smaller than 32 mm. Therefore, our glycine/PVP films can show high flexibility. In addition, the soft and continuous PVP could effectively dissipate the mechanical impacts on the fragile glycine crystals. Meanwhile, the nano-scale glycine crystals seamlessly encapsulated by PVP also largely minimized the defective spots of the film and are favorable for their flexibility. We have added the description in the Note S4 in the revised supplementary information.

Note S5. Melting point depression effects of *β*-glycine nanosized crystals

Within the critical size range, the thermotropic properties of crystals are anticipated to exhibit a strong correlation with size, as the magnitudes of volume free energy and surface free energy are comparable. This phenomenon has been observed in various materials such as ice, metals, and organic solids, where significant shifts in melting points and enthalpies of fusion have been observed due to confinement within crystals. The unique crystal properties of these materials have been revealed through the process of crystallization within nanoporous container. Nanoconfinement plays a crucial role in altering the thermotropic properties of crystals. This effect can be mathematically described by the classical Gibbs-Thomson equation:

$$\frac{\Delta T_{melt}}{T_{bulk}} = -\frac{2M}{\Delta H \rho r}\gamma \cos\theta \qquad \text{(S13)}$$

where M refers to the molecular mass of the compound composing the particle, ρ represents the particle density, r denotes the particle radius (assuming a spherical shape), γ represents the specific interfacial energy (surface tension) between the solid phase and the surrounding fluid, θ denotes the interfacial angle between the condensed phase and any nucleated phase, ΔH represents the molar heat of fusion of the bulk condensed phase, $T_{melt}(r)$ represents the melting temperature of the condensed phase with radius r, and T_{bulk} corresponds to the melting temperature of the condensed phase in its bulk state.

The Gibbs-Thomson equation predicts that there is a linear inverse relationship between the change in melting temperature (ΔT_{melt}) and the crystal size (r), assuming that all other parameters remain constant and independent of particle size. In the context of homogeneous nucleation, the contact angle (θ) is often assumed to be 180°. The impact of size on polymorphism can be described by a simplified version of the Gibbs-Thomson equation as follows:

$$\frac{\Delta T_{melt}}{T_{bulk}} = -\frac{2M}{\Delta H \rho r}\gamma \qquad \text{(S14)}$$

According to this equation, the melting point is expected to decrease as the size of the crystals is reduced. Consequently, it can be inferred that β-glycine nanocrystals-PVP composites would exhibit distinct thermotropic properties compared to their bulk counterparts. This inference aligns with the experimental results we have obtained.

Note S6. Finite element method (FEM) simulations

A three-dimensional multi-field coupling simulation was conducted using COMSOL Multiphysics 6.1 to investigate the behavior of the transcutaneous ultrasound energy harvester. A square-shaped piezoelectric thin film (40×40 mm^2) was embedded within a cylindrical tissue phantom (i.e., sound domain) with a radius of 100 mm and a thickness of 90 mm. To simulate the external ultrasound transducer, a circular sound source with a radius of 25 mm was positioned at the center of the domain's upper surface. The 1-mm thick piezoelectric film was implanted beneath the sound source at a depth of 17 mm (Fig. S26).

This simulation employed three physical modules: Pressure Acoustic (Frequency Domain), Solid Mechanics, and Electrostatics. The ultrasound waves, transmitted from the external ultrasound transducer, propagated within the tissue at a frequency of 40 kHz, oscillating the tissue particles as well as the inserted flexible membrane. As a consequence of the sound-induced compression and tension, the piezoelectric membrane deformed, leading to a periodic alteration of the electric potential distribution along its thickness (Video. 4). The material properties of the tissue phantom in this simulation were based on previous empirical data (*50*), which closely resembled the properties of actual human tissues (*51*), as illustrated in Table S8. The mechanical and piezoelectrical parameters of the bio-organic films were referenced from the measured values in this study.

The presence of the Gly-PUEH within tissue affects the wave front of ultrasound due to the mismatch in acoustic impedance. To further investigate the reliability and safety of the system, we analyzed the acoustic pressure and sound intensity on the perpendicular cross-section of the tissue phantom, specifically focusing on the region where the membrane section was removed (Fig. S27). When the ultrasound source emits waves at an acoustic pressure of 100 kPa, the sound energy exhibits an effective penetration depth of approximately 50 mm. This indicates that the bio-organic film efficiently receives and responds to the acoustic oscillation. The ultrasonic wavefront, in return, is distorted by the presence of this thin obstacle, as depicted through the Acoustic-Structure Boundary in the simulation. Furthermore, it is worth noting that this system demonstrates exceptional safety characteristics as it ensures that the sound intensity within the tissue remains below 720 mW/cm^2, aligning with the safety regulations for ultrasound diagnoses and therapies set by the United States (U.S.) Food and Drug Administration (FDA).

To delve deeper into the impact of piezoelectric particles on the electric potential of the film, a two-dimensional multi-field coupling simulation was carried out at the micro level. Given the substantial dimension disparity between the grain diameter (~1 μm) and the film thickness (~1 mm), an accurate microstructure representation would result in a highly time-consuming simulation. Consequently, a schematic and partial cross-section of the thin film was employed as a simplified approximation (Fig. S28). The rectangular region contains multiple disjoint circles corresponding to the circular cross-sections of piezoelectric grains, which are generated using the Random Circle codes in MATLAB. The diameter and interstice of these circles can be adjusted to approximate the spatial distribution of particles within the film closely. The mechanical and piezoelectrical parameters of both the particles and the encapsulating polymer are determined by referencing the measured values in this study. Under the 40 kHz ultrasound energy, the film and internal piezoelectric particles experience compression and stretching in the thickness direction, leading to a periodically changing electric potential distribution within the film at the same frequency (Movie. 5).

Supplementary Figures

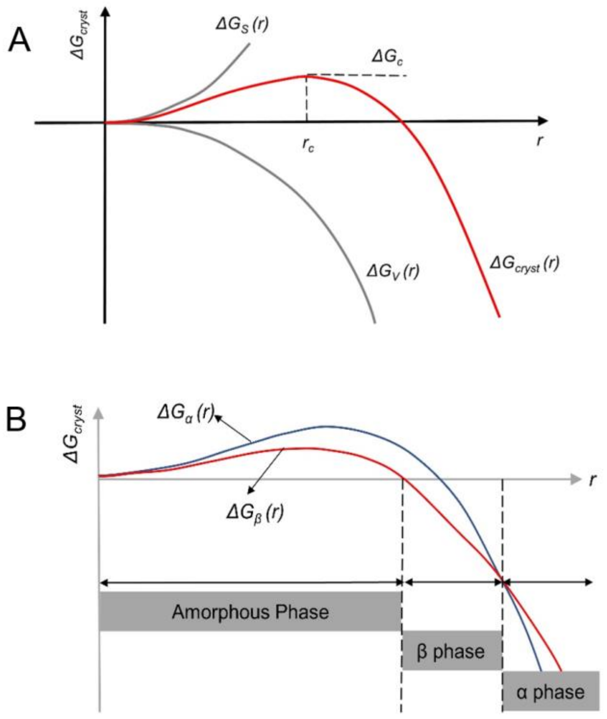

Fig. S1. Free energy profile of a growing crystal nucleus. (**A**) Illustration of the free energy (ΔG_{cryst}) profile of a growing crystal nucleus as a function of crystal radius, r. The energy profile results from the sum of the favorable volume free energy, ΔG_V, and the surface free energy associated with forming an interface between the new phase and its surrounding medium, ΔG_S. The profile passes through a maximum value of ΔG_{cryst} at the critical radius, r_c. (**B**) Depiction of the free energy profiles that depend on size for two competing nuclei, namely α-glycine and β-glycine.

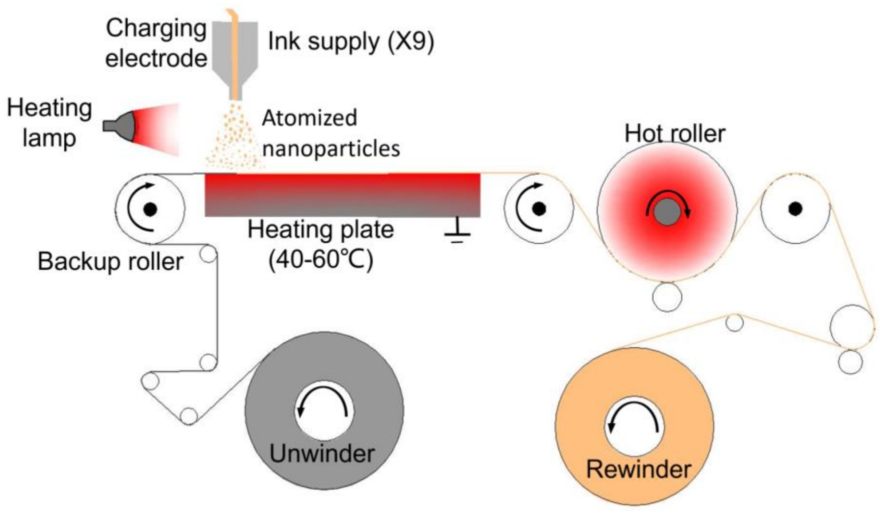

Fig. S2. Schematic of the TEA printer.

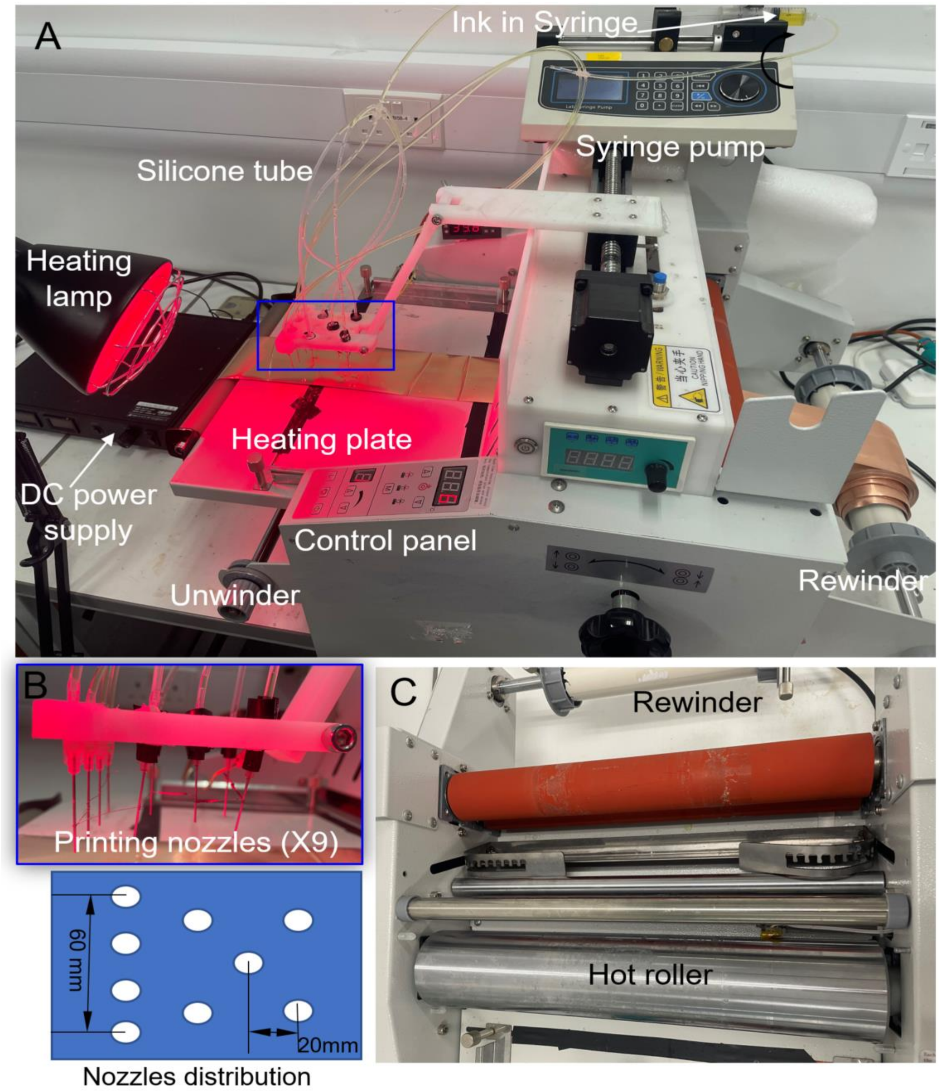

Fig. S3. Photos of the TEA printer.

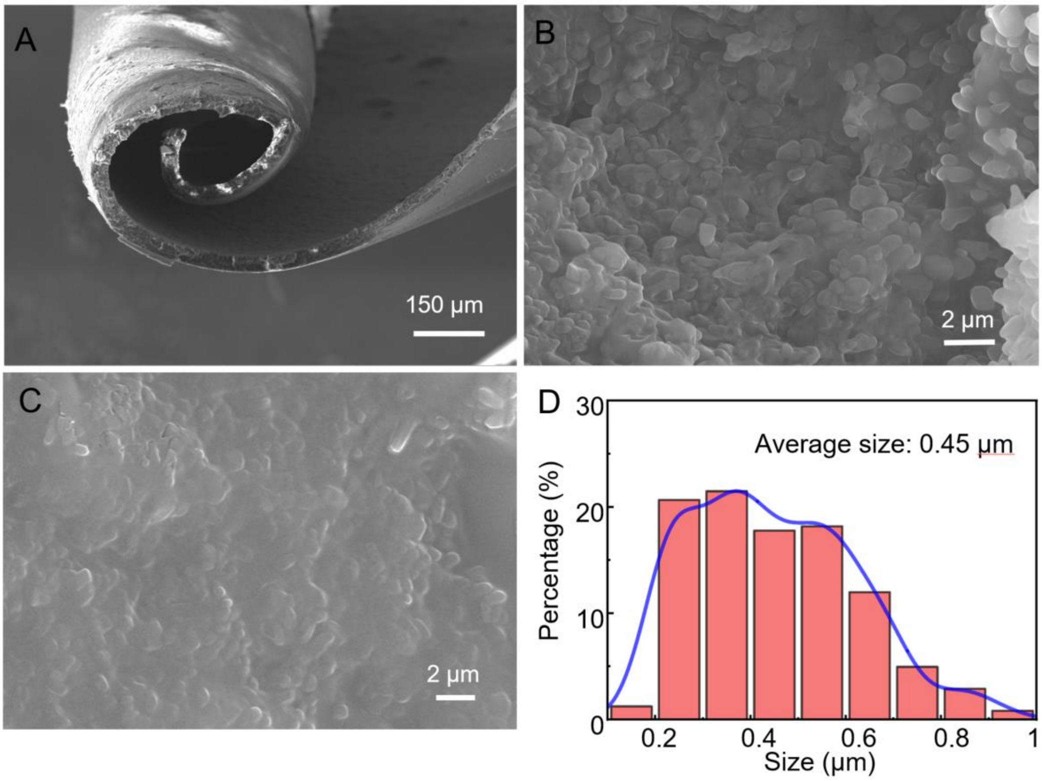

Fig. S4. SEM images and corresponding grain size distribution result of glycine/PVP films. (A) Flexible glycine/PVP films. **(B)** Cross-sectional and **(C)** surface topography SEM images showing the compact nanosized grains of the uniform and continuous films. **(D)** Grain size distribution of glycine nanograins.

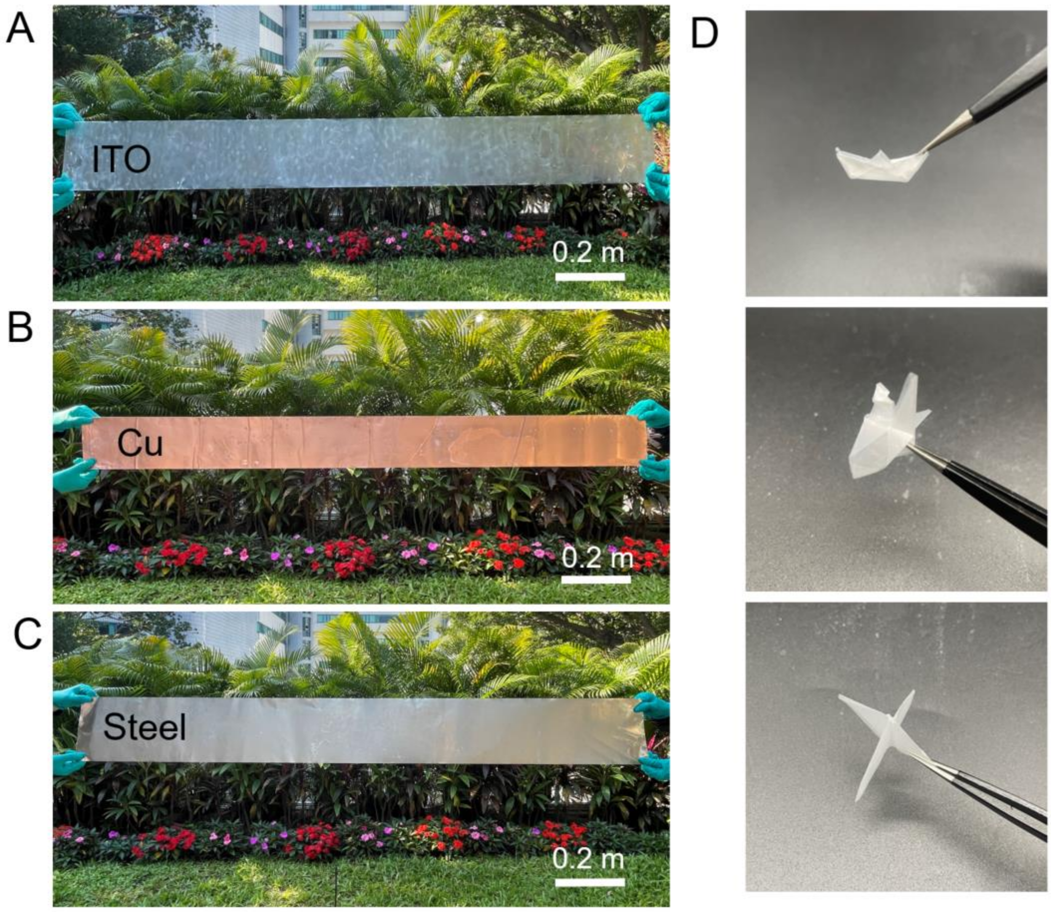

Fig. S5. Photograph of the glycine/PVP films. (A, B, C) Glycine/PVP films deposited on different substrates (ITO, copper and steel). **(D)** Some photographs showing flexibility of glycine/PVP films.

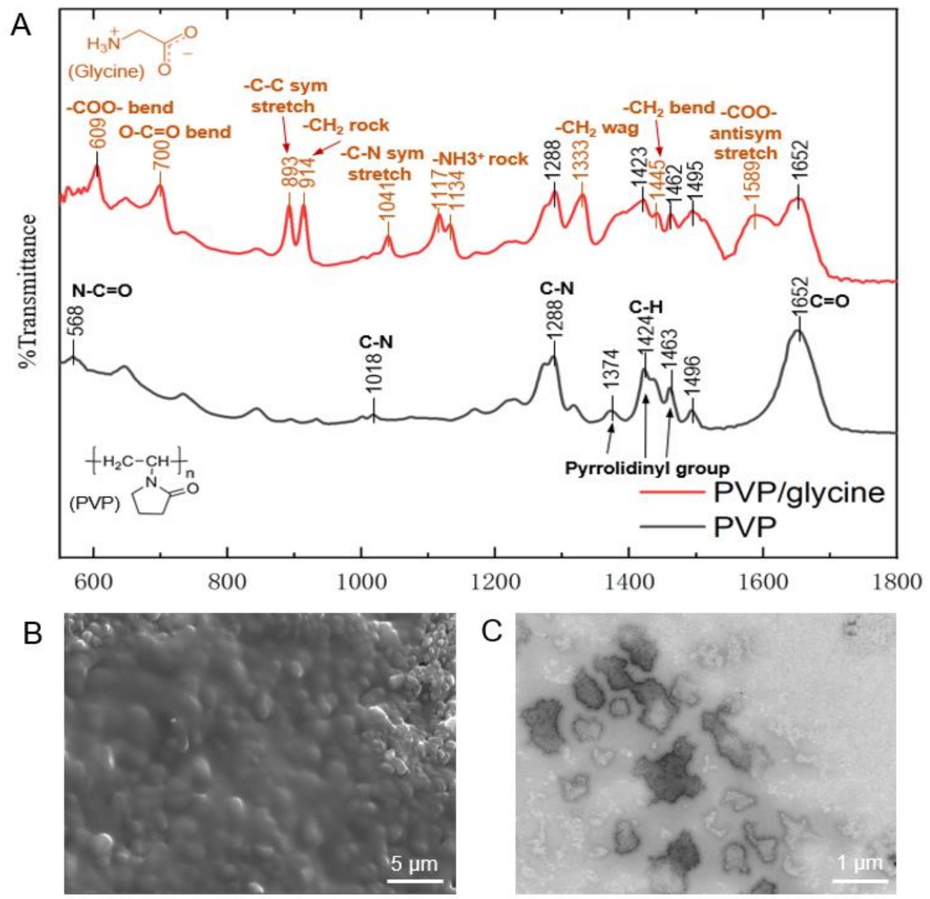

Fig. S6. Interfacial interaction analysis between the glycine particles and the PVP. (A) FTIR spectra of glycine/PVP films and pure PVP films. **(B)** SEM image of glycine crystals within PVP polymer. **(C)** TEM image of glycine crystals within PVP polymer.

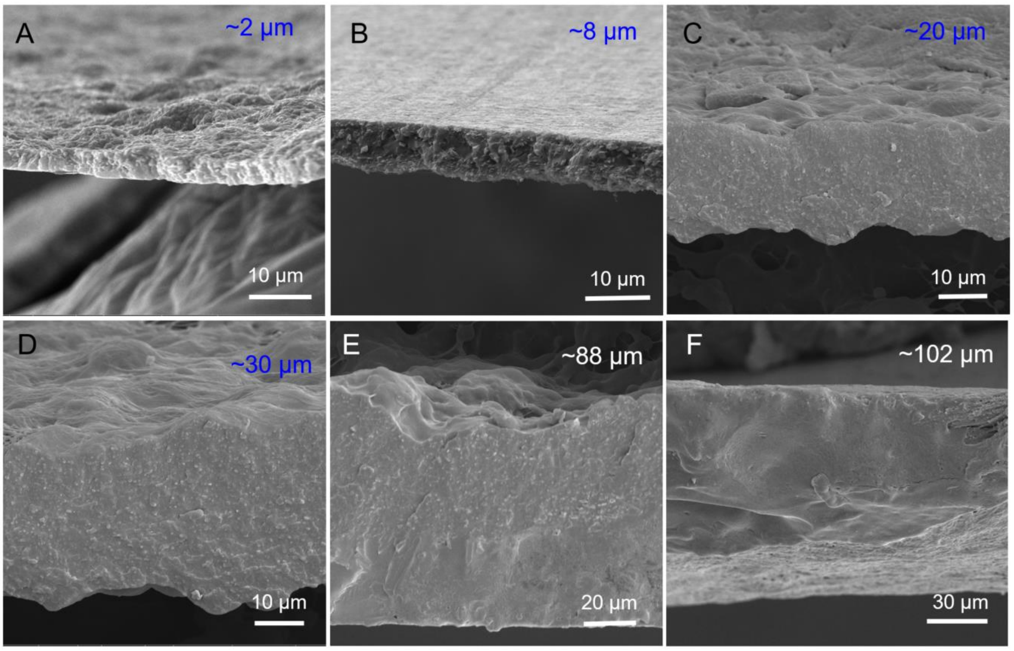

Fig. S7. Glycine/PVP films with different thickness (from ~2 to ~100 μm).

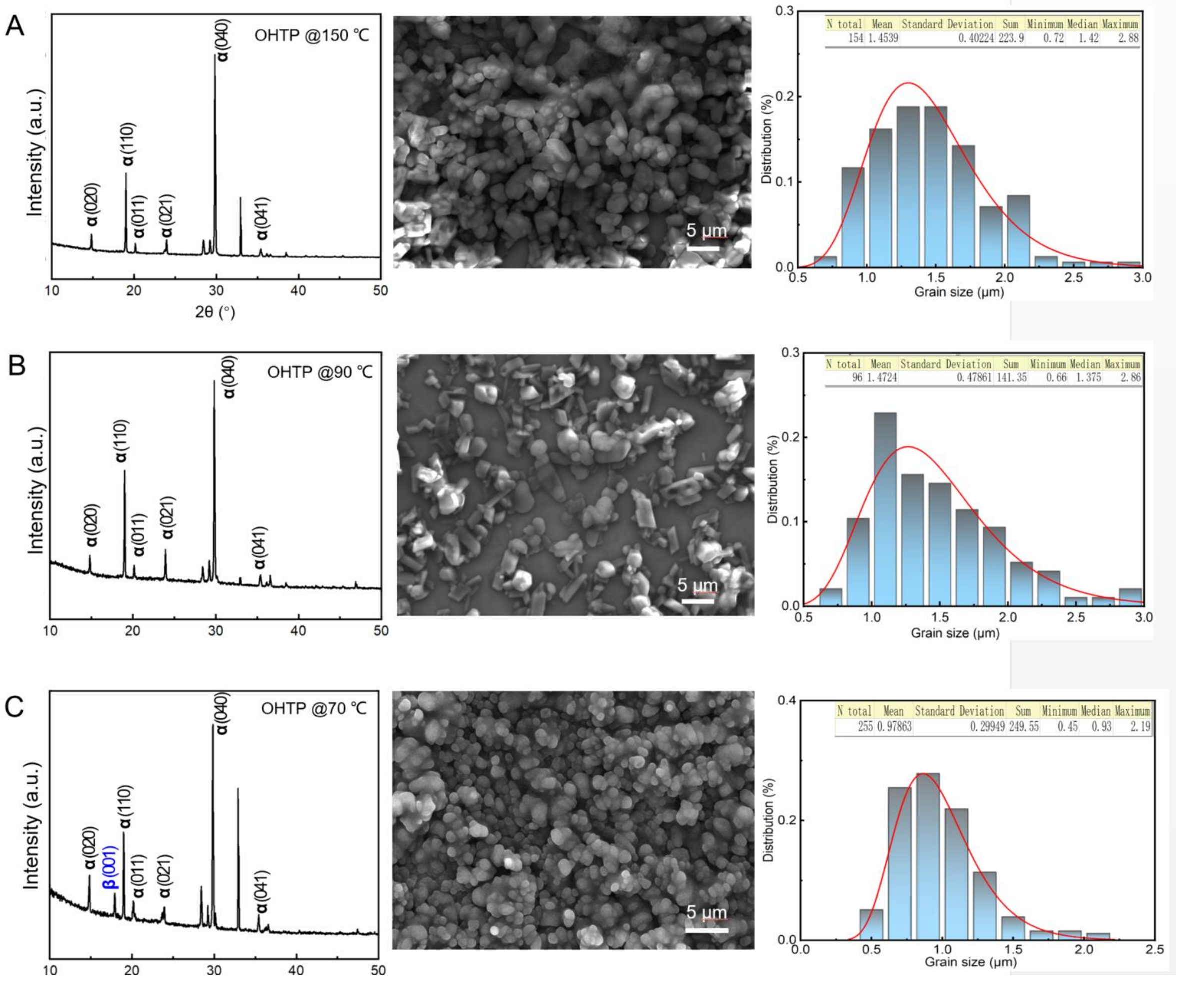

Fig. S8. XRD spectrum, SEM image of deposited grains, and grain size distribution for glycine/PVP films fabricated at different temperatures. (**A**) 150 °C. (**B**) 90 °C. (**C**) 70 °C.

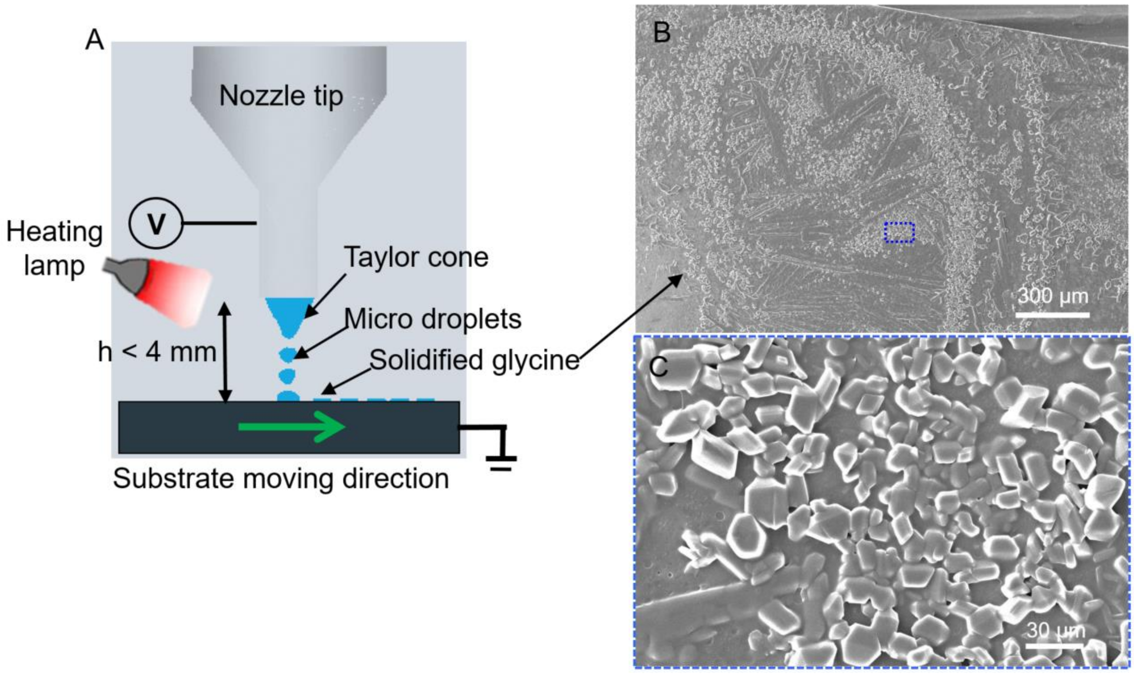

Fig. S9. Glycine microdots fabricated by electrohydrodynamic focused printing. (**A**) Schematic of the electrohydrodynamic focused printing to fabricate glycine microdots. (**B, C**) Surface topography SEM images of the glycine microdots.

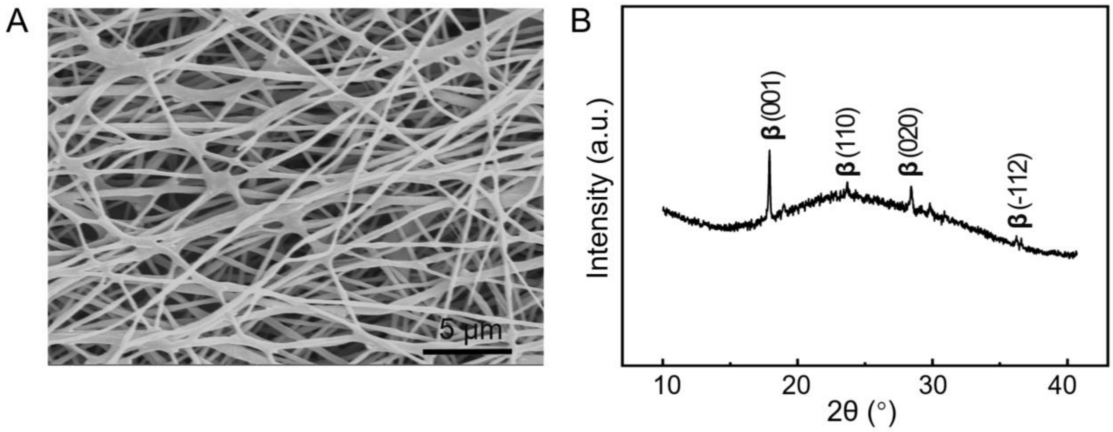

Fig. S10. Glycine/PVP nanofibers fabricated by electrospinning. (**A**) Surface topography SEM image and (**B**) XRD spectrum of the glycine/PVP nanofibers. The glycine nanofibers are obtained by electrospinning. PVP/glycine precursor aqueous solution containing 20 % PVP and glycine solution in a 2:1 weight ratio is used for electrospinning. The molecules tend to align with their molecular dipole moments parallel to the fiber plane and aligned with the fiber longest axis.

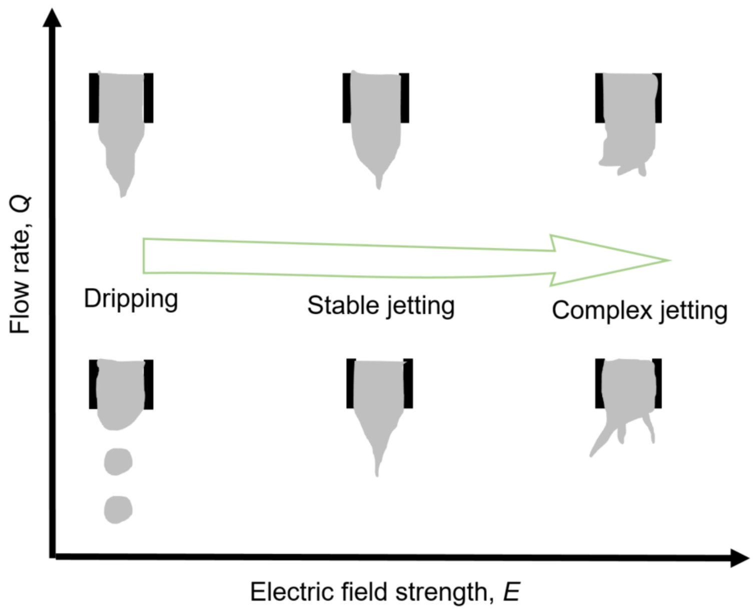

Fig. S11. Formation of different fluid modes, depending on the electric field strength and flow rate.

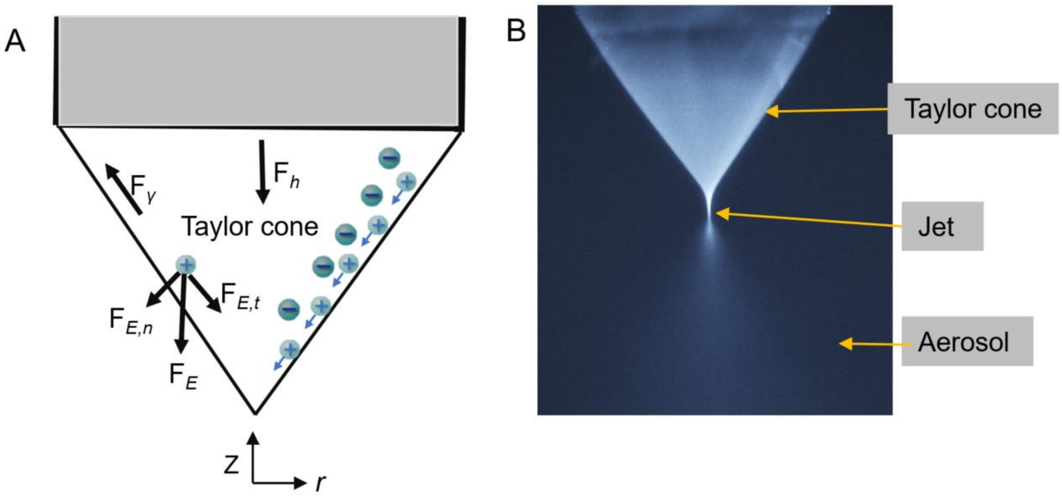

Fig. S12. Electrohydrodynamically induced jet of TEA printing. (**A**) Schematic showing the forces acting on the fluid surface during TEA printing. (**B**) Photograph of the electrohydrodynamically induced jet.

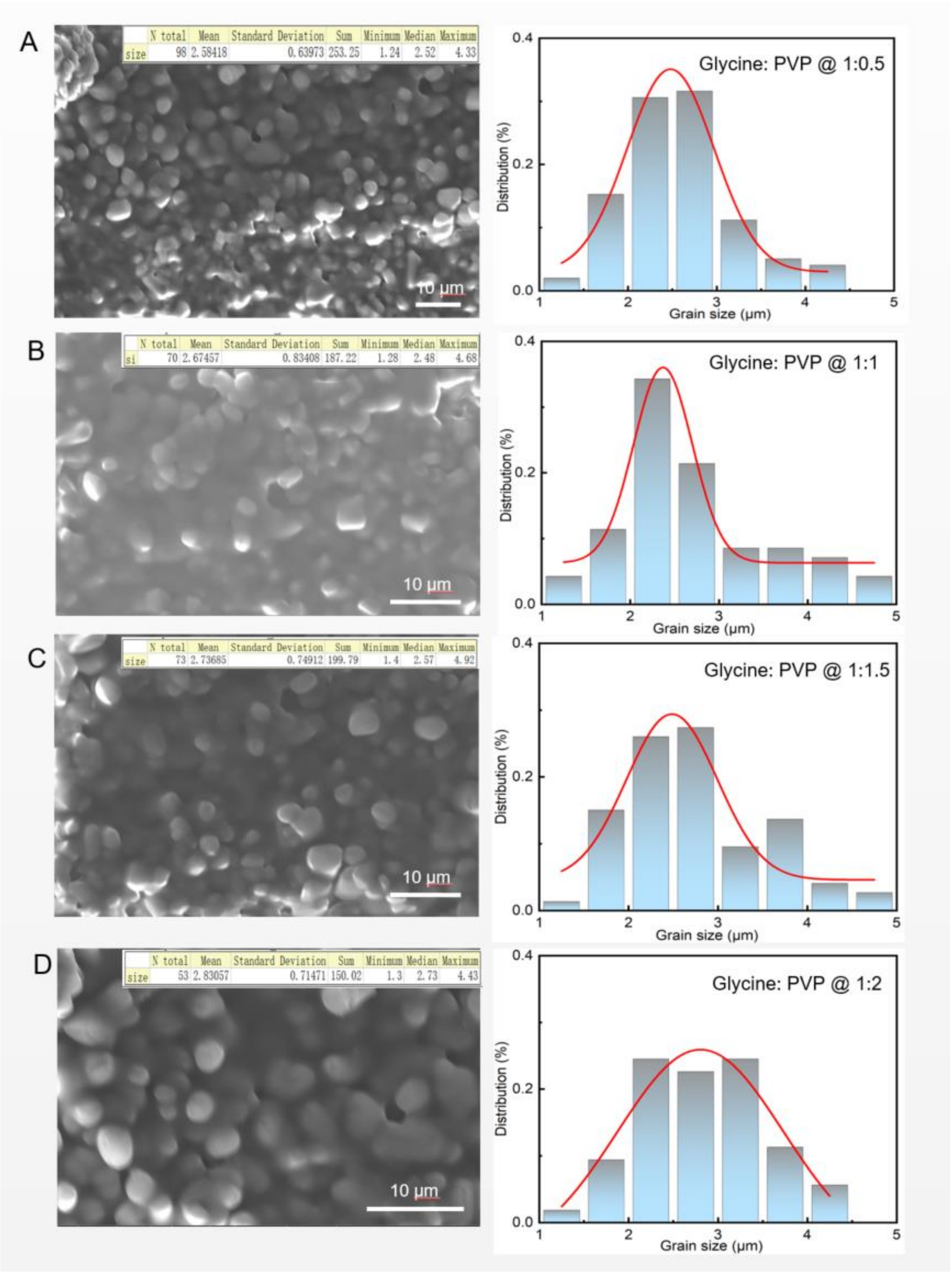

Fig. S13. SEM image of deposited grains, and grain size distribution for glycine/PVP films fabricated at 25 °C using glycine/PVP ink with different mixing rate. (**A**) glycine/PVP (1:0.5). (**B**) glycine/PVP (1:1). (**C**) glycine/PVP (1:1.5). (**D**) glycine/PVP (1:2).

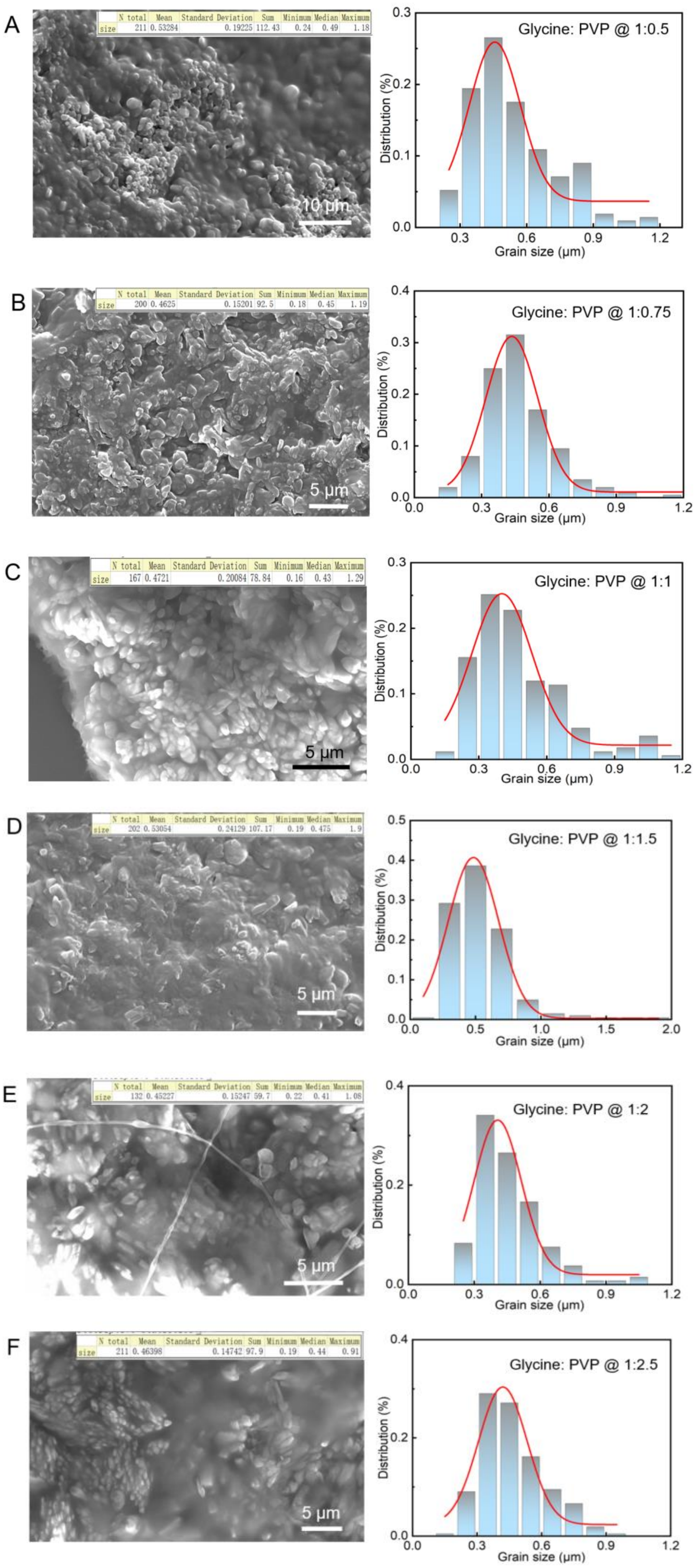

Fig. S14. SEM image of deposited grains, and grain size distribution for glycine/PVP films fabricated at 60 °C using glycine/PVP ink with different mixing rate. (A) 1:0.5; (B) 1:0.75; (C) 1:1; (D) 1:1.5; (E) 1:2; (F) 1:2.5.

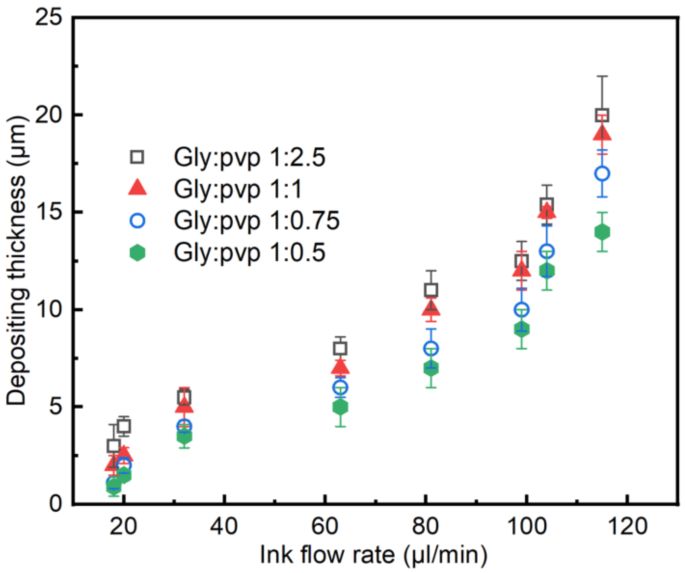

Fig. S15. Deposited film thickness versus flow rate of inks with different glycine-to-PVP mixing rate. Error bars represent s.d. from five experimental results.

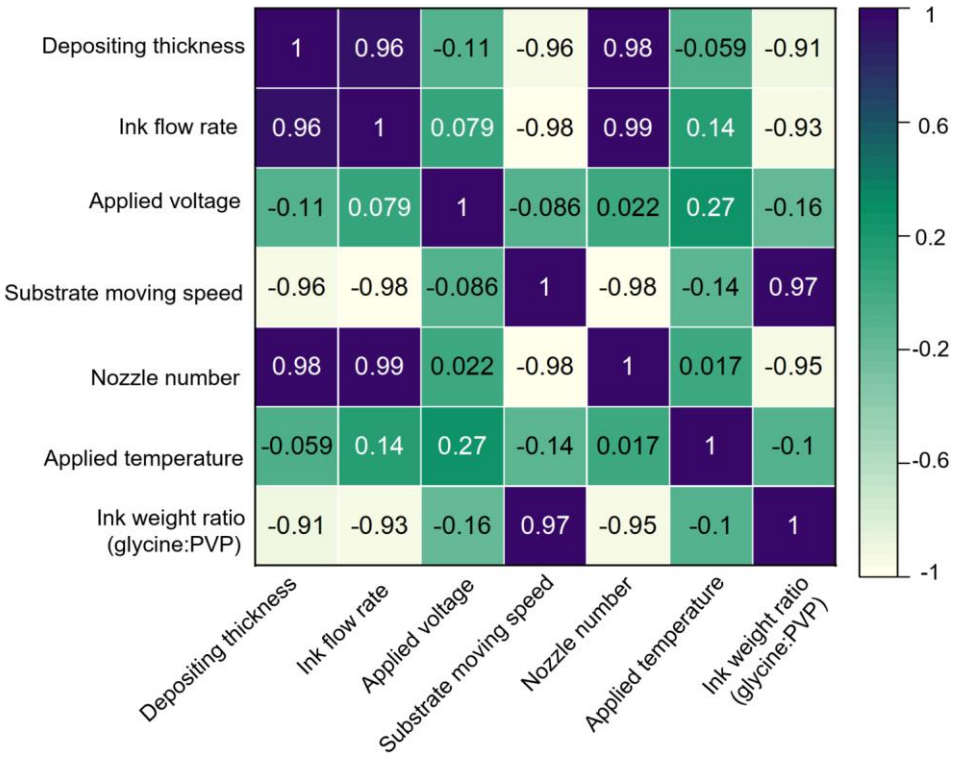

Fig. S16. Correlation matrix of deposition thickness versus different printing parameters

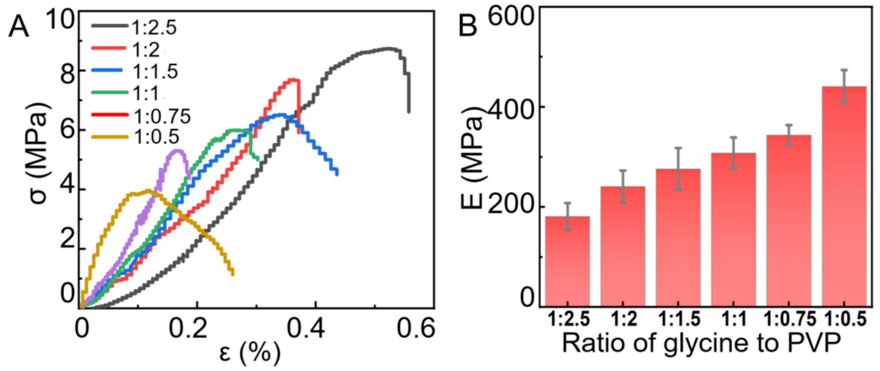

Fig. S17. Stress-strain curves and elastic moduli of glycine/PVP films. (**A**) Stress-strain curves of as-prepared glycine/PVP films with different composition ratios. σ, tress; ε, strain. (**B**) Elastic moduli of the films calculated from the stress-strain curves in (A).

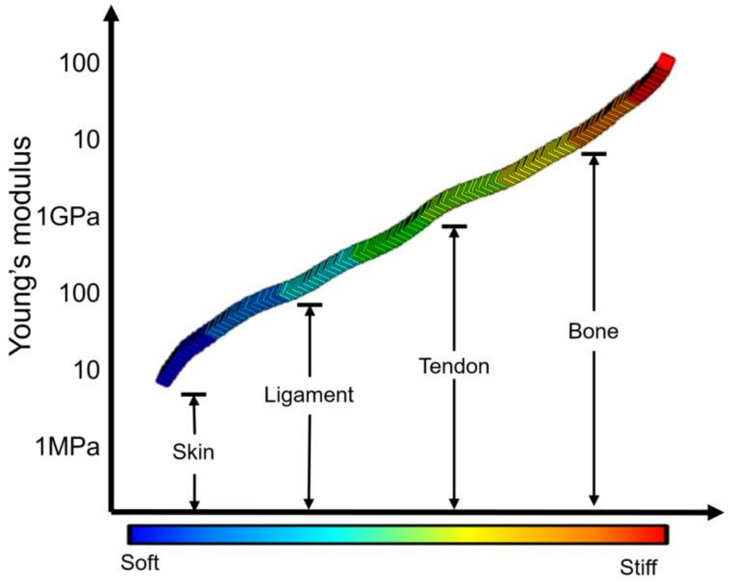

Fig. S18. Young's modulus of the glycine/PVP films in comparison with that of some biomaterials (*52*).

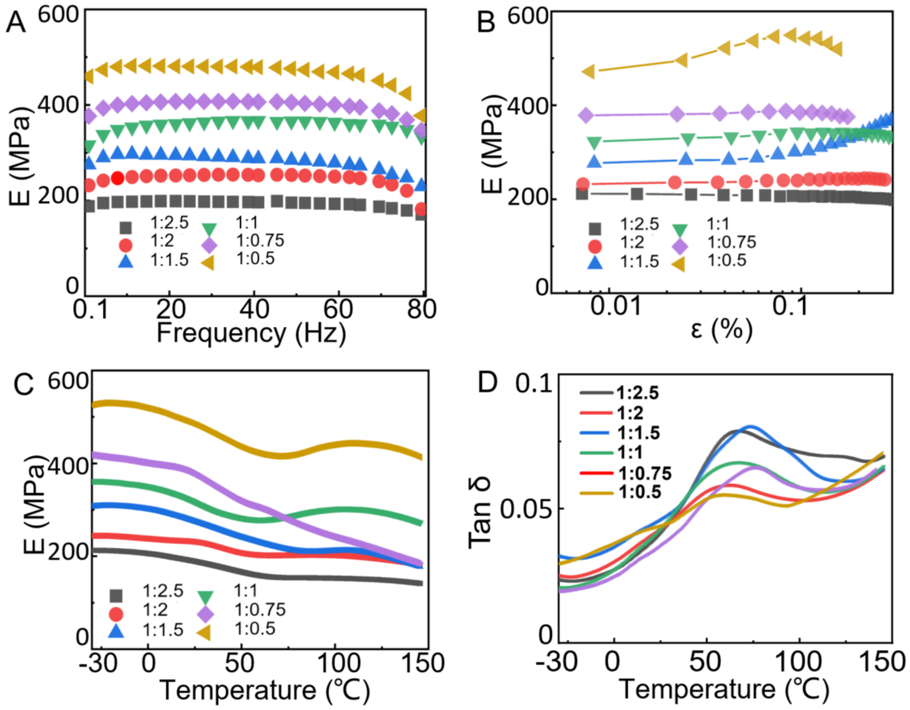

Fig. S19. **Mechanical properties of glycine/PVP films.** (**A**) Dynamic mechanical analysis of as-prepared glycine/PVP films in the frequency sweep mode from 0.1 to 80 Hz. (**B**) Dynamic mechanical analysis of as-prepared glycine/PVP films in the strain sweep mode from 0.01 to 0.2% strain at a constant frequency of 1 Hz. (**C, D**) Dynamic mechanical analysis of as-prepared glycine/PVP films in the temperature sweep mode from -30 to 150°C.

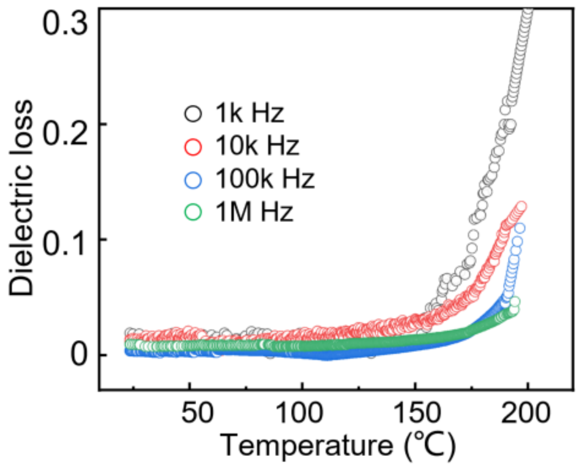

Fig. S20. **Dielectric loss as a function of temperature for glycine/PVP films.**

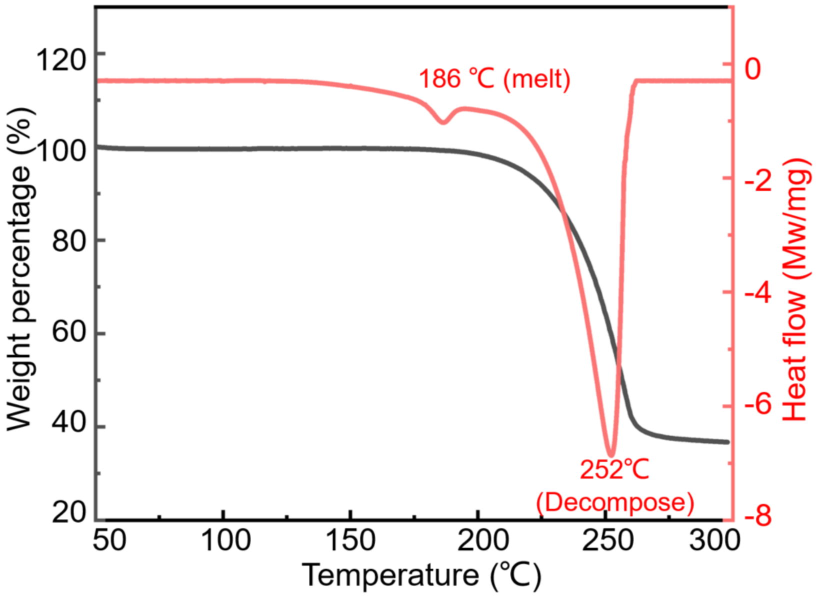

Fig. S21. **DSC (red curve) and TGA (black curve) results of the as-prepared glycine/PVP films.**

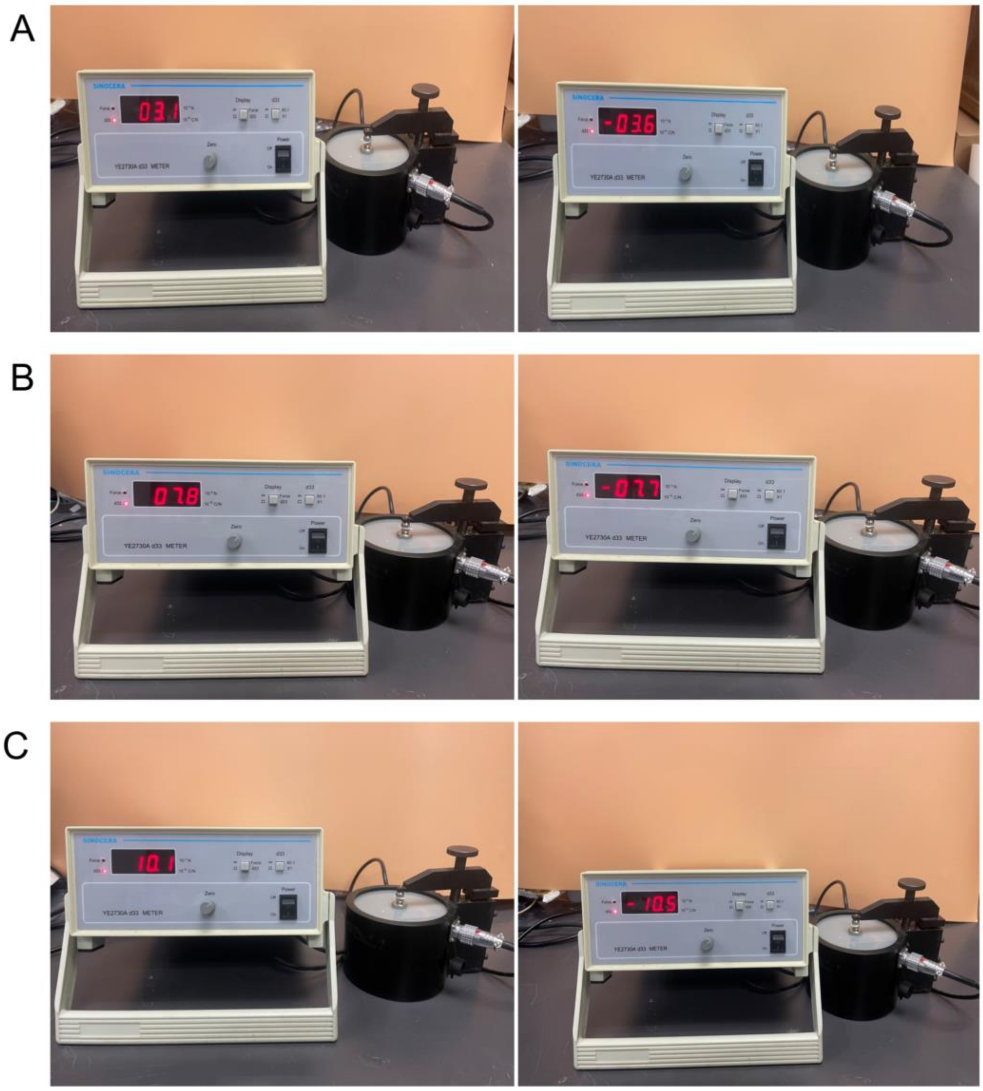

Fig. S22. **Macroscopic piezoelectricity of the glycine/PVP films with different glycine-to-PVP mixing ratio.** (**A**) 1:1.5, (**B**) 1:0.75, (**C**) 1:0.5.

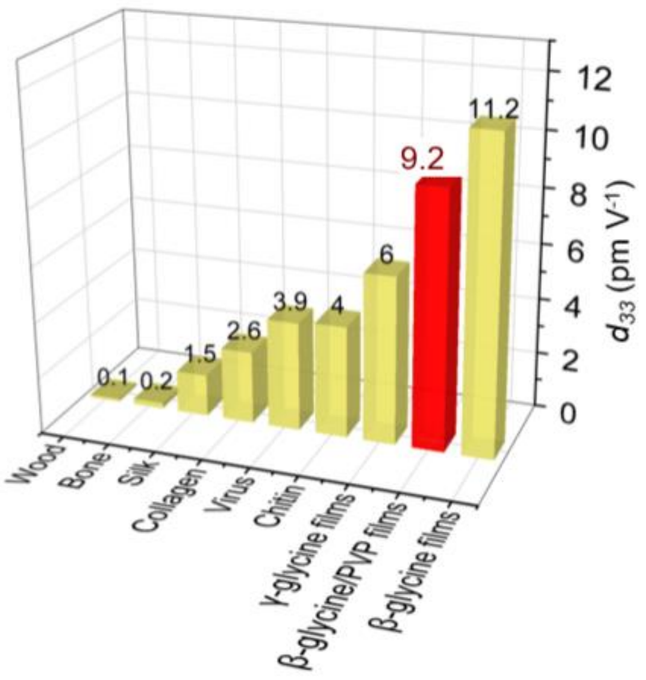

Fig. S23. Piezoelectric coefficient of the as-prepared glycine/PVP (1:1) films compared with other bio-organic piezoelectric materials.

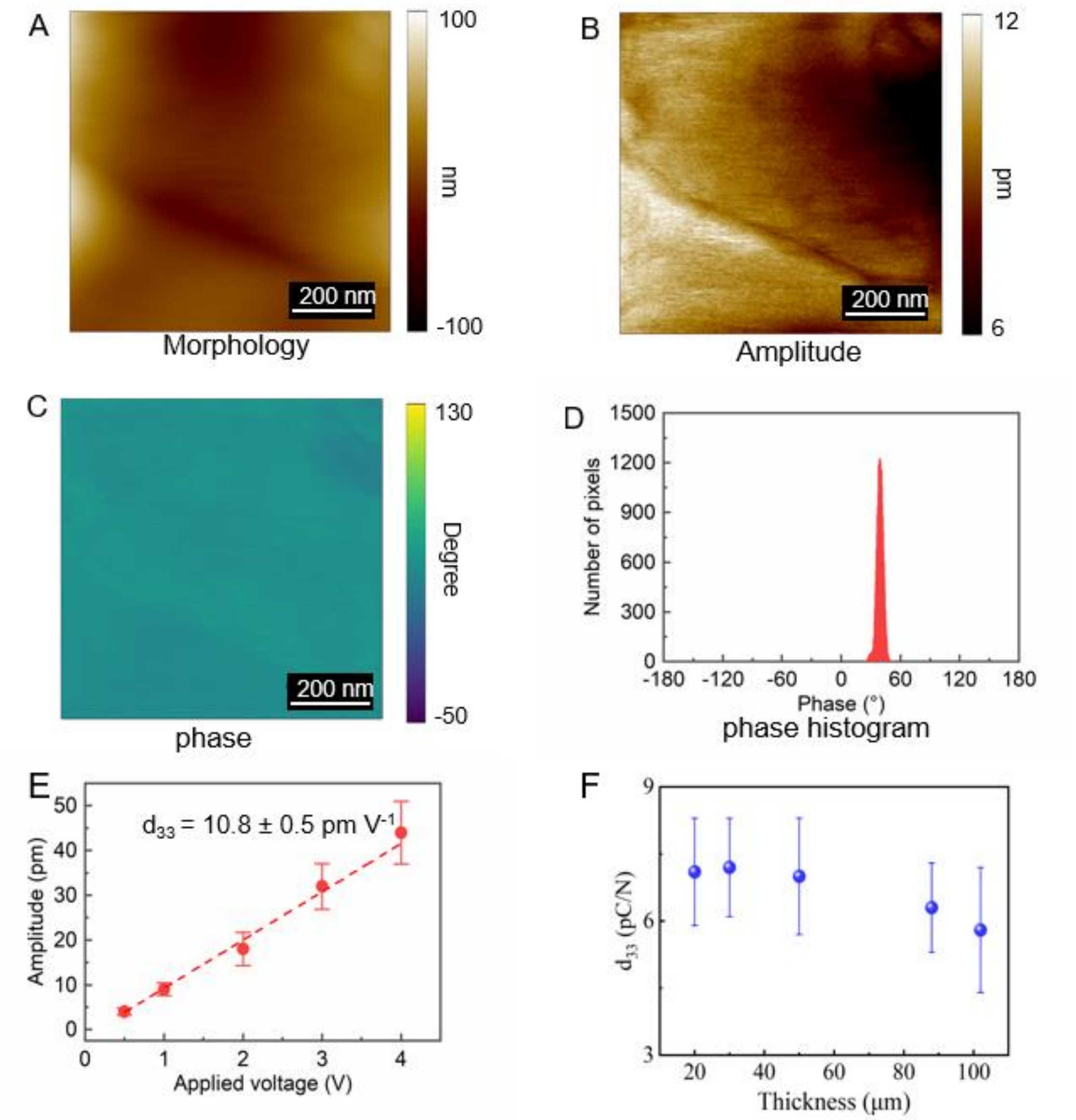

Fig. S24. PFM measurements of glycine/PVP films. (**A**) 3D AFM topography image of the as-deposited glycine/PVP films. (**B**) The PFM OOP amplitude mapping overlaid on the 3D topography of as-prepared glycine/PVP (1:1) films in a 1 × 1 μm^2 area. The applied AC voltage is 1 V. (**C**) The PFM OOP phase mapping overlaid on the 3D topography. (**D**) Histogram of a large-area PFM OOP phase mapping in (C) showing that the films are dominated by domains with the unique polarization direction. (**E**) Linear dependence of PFM amplitudes on the applied AC voltage. The error bar denotes the standard deviation. (**F**) d_{33} coefficients of glycine/PVP films (1:1) with different thickness.

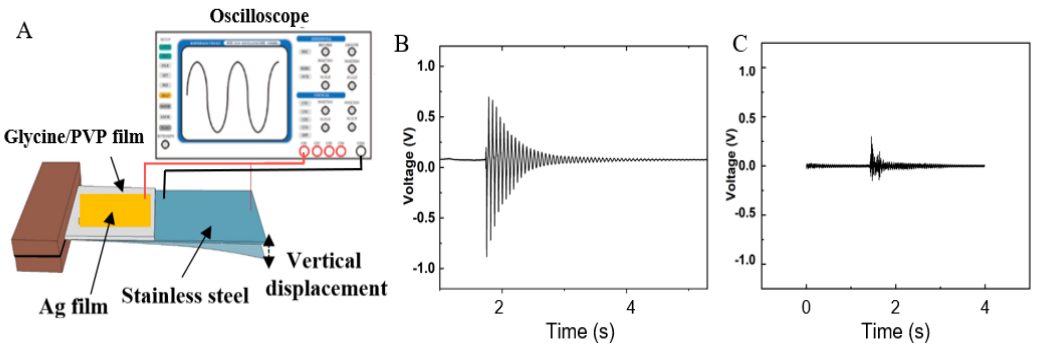

Fig. S25. Voltage signal of the cantilever system based on glycine/PVP film. (A) Schematic of the cantilever system to characterize the piezoelectric performance of the glycine/PVP films. (B) Voltage signal of the cantilever system based on a ~ 10 μm thick glycine/PVP film. Its open-circuit voltage output corresponds to the damped and attenuated vibration, and a maximum voltage of nearly 0.7 V. (C) Voltage signal of the cantilever system based on the control device (pure PVP film).

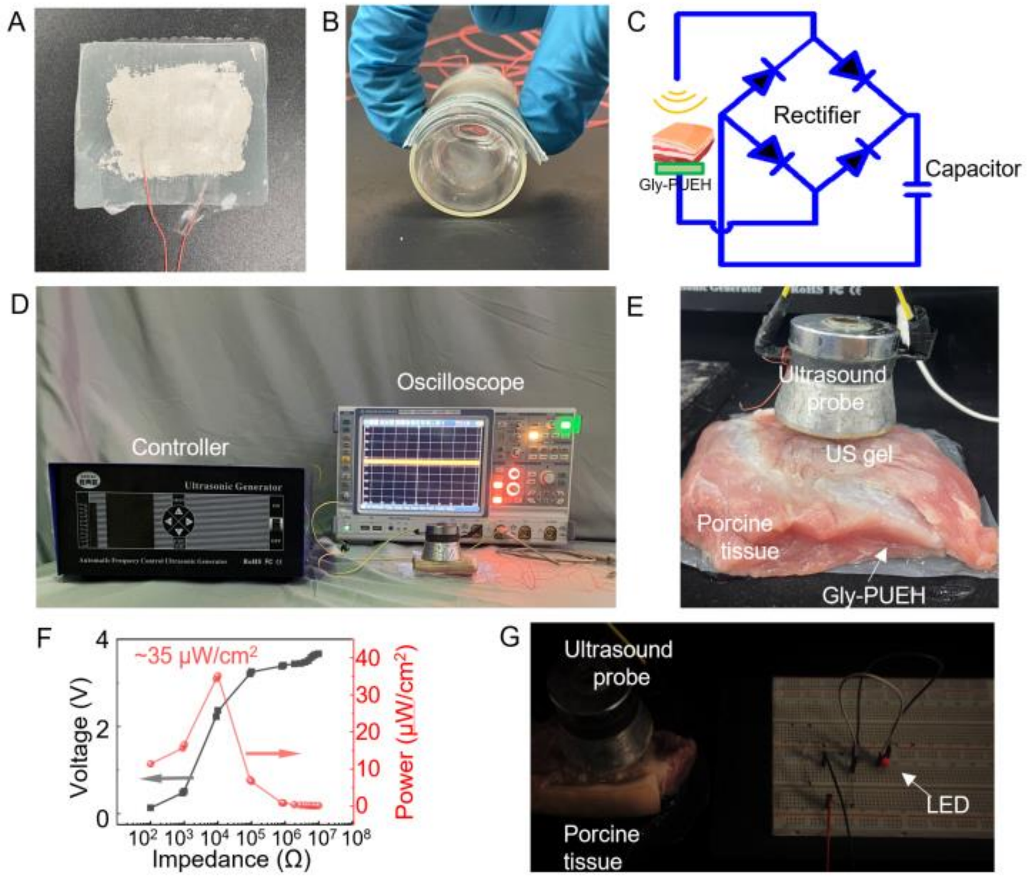

Fig. S26. Ex-vivo characterization and output signals of the Gly-PUEH. (**A, B**) Photograph of the Gly-PUEH bent on a beaker, showing its high flexibility. (**C**) Schematic diagram of the full-bridge rectifier and storage circuit. (**D, E**) Ex-vivo characterization of the Gly-PUEH implanted inside the porcine tissue under ultrasound. (**F**) Output voltage and power density depend on the resistance. The maximum power is ~35 μW/cm^2. (**G**) Optical photograph of one red LED lit up by the Gly-PUEH.

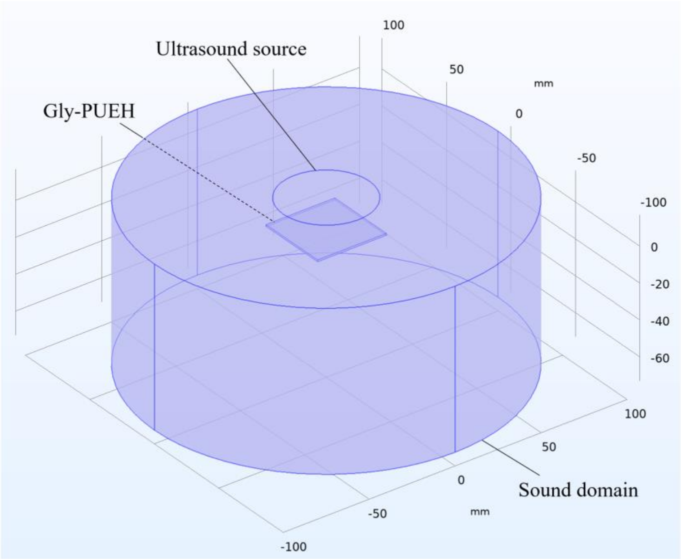

Fig. S27. Geometric model of the sound domain

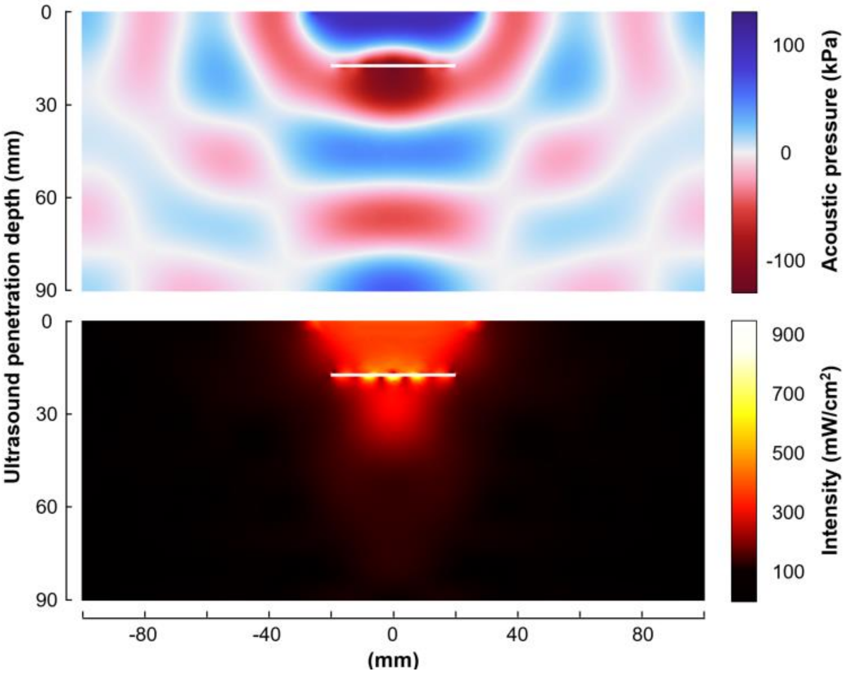

Fig. S28. Simulated acoustic pressure (top) and the sound intensity (bottom) on the perpendicular cross-section of the sound domain.

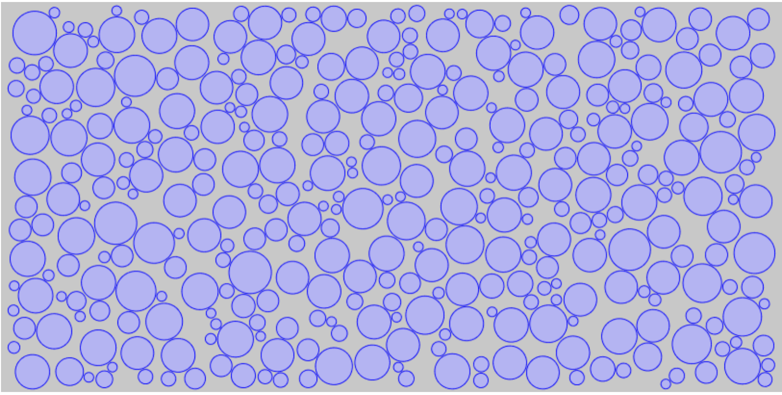

Fig. S29. Schematic cross-section of the thin film containing piezoelectric particles.

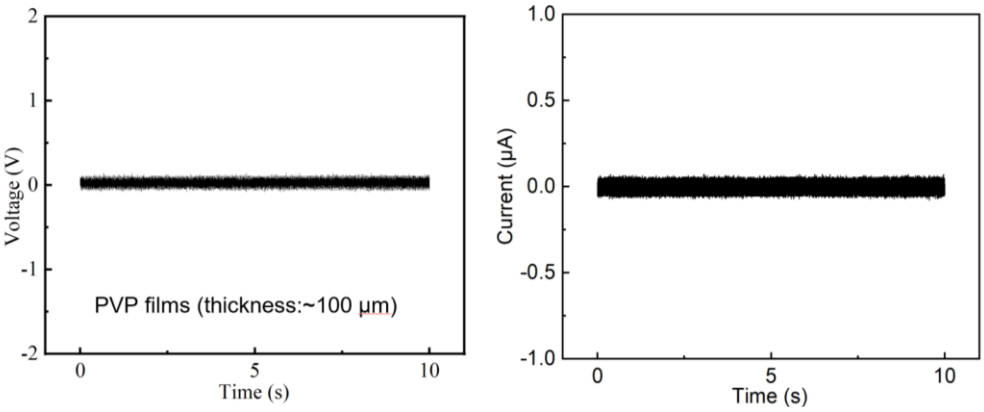

Fig. S30. Open-circuit voltage and current of the control device (PVP film), which is implanted inside the porcine tissue under ultrasound.

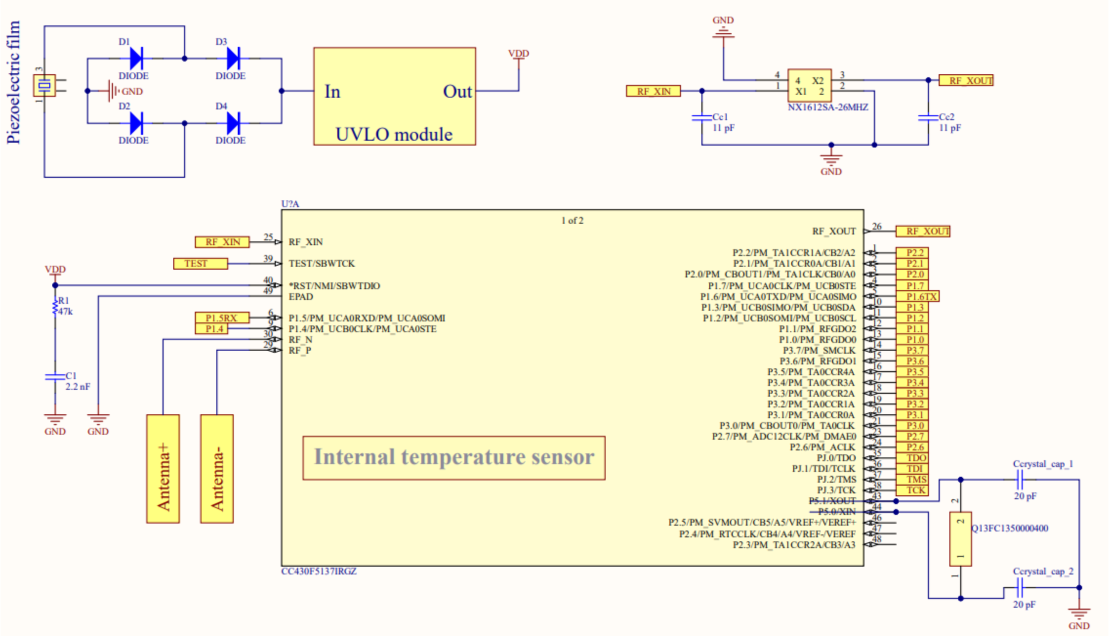

Fig. S31. Circuit diagram of the wireless communication configuration with a wireless temperature sensor supplied by the Gly-PUEH ex vivo.

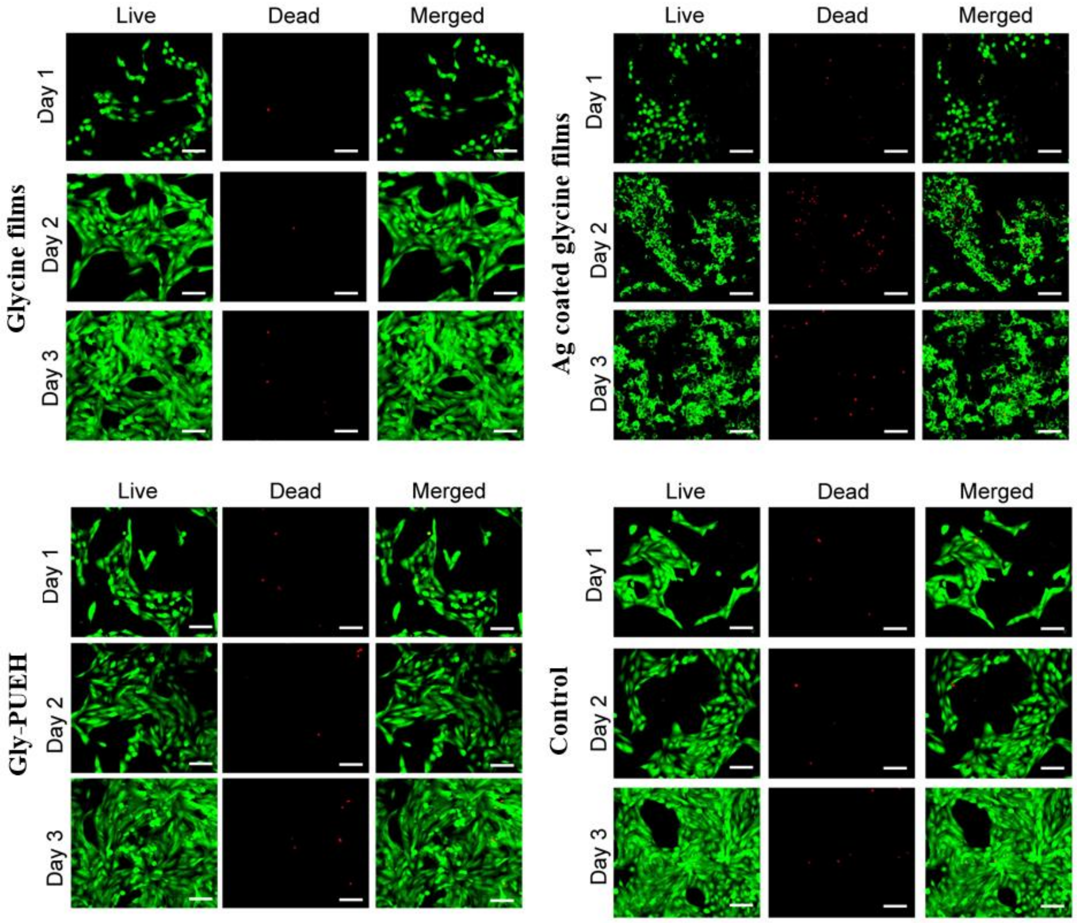

Fig. S32 Fluorescent images of the myofibroblasts cultured with different sample extracts on days 1, 3, and 5. Scale bar, 100 µm.

Supplementary Tables

Table S1. The major components of the TEA printer.

Item	Manufacturer	Amount
R2R coating system	Da Yin Jia Co., Ltd	1
Micro pump	Beijing Sichuang Technology Instrument Co., Ltd	1
DC power supply	Wendong High Voltage Power Supply Co., Ltd	1
Printing nozzle	Home-made	9
Temperature controller	Xiaochuangxin Co., Ltd	1
Y motion stage controller	Huizhi Electric Textile Co., Ltd	1
Digital camera	Industrial Vision Technology Co., Ltd	1

Table S2. List of depositing speeds of representative manufacturing techniques for piezoelectric films, which are plotted in Fig. 1H. Polymethyl methacrylate (PMMA); Poly(ethylene naphthalate) (PEN); Aromatic dipeptide nanotubes (ADNTs); Poly(γ-benzyl α , L-glutamate) (PBLG); poly(ethersulfone) (PES).

**Materials**	**Fabricating strategy**	**Substrates**	**Thickness (μm)**	**Fabricating speeds （$\mu m^3\ s^{-1}$）**
β-glycine/chitosan (*53*)	Solution casting	polystyrene petri dish	~38	$6.2\times10^5 – 1.24\times10^6$
γ-glycine (*37*)	Solution casting	Silicon	~16	3.43×10^7
γ-glycine (*54*)	Solution casting	PMMA、petri dish	~16	5.1×10^6
Diphenylalanine (*38*)	Electric field assisted casting	Gold-coated silicon	~60	4.3×10^5
Diphenylalanine (*55*)	Dip-coating	Au/Cr-coated PEN	~0.2	5×10^3
Diphenylalanine (*56*)	Spin-coating	Silicon	~0.05-~0.4	~2.3×10^2-~1.8×10^3
Diphenylalanine (*57*)	Epitaxial growth on seed film	Silicon	~50	1.84×10^5
Diphenylalanine (*58*)	High temperature aniline vapor aging	Silicon	10	2.3×10^4
PBLG (*59*)	Electrospinning	Aluminum foil	~40	＜4.7×10^7
Silk fibroin (*60*)	Electrospinning		＜100	1.67×10^3、5×10^3、1.3×10^4
**β-glycine/PVP**	**This work**	**ITO、Copper foil、steel**	**~1-100**	**~3.3×10^8**

Table S3. Physical properties of glycine/PVP inks. Experimental physical properties are measured at 25 °C.

Ratio (glycine to PVP)	Density (10^3 kg m^{-3})	Surface tension (10^{-3} N m^{-1})	Relative permittivity	Electrical conductivity (S m^{-1})
1:2.5	1.089	78.9	9.3	0.043
1:2	1.028	78.3	8.8	0.045
1:1.5	0.999	77.8	8.6	0.047
1:1	0.996	77.8	8.1	0.053
1:0.75	0.988	77.4	7.2	0.055
1:0.5	0.983	77.1	6.2	0.058

Table S4. Optimized parameters of TEA printing.

Parameter	Value
Depositing speed (mm s^{-1})	1~30
Distance between disc to substrate (mm)	4~10
Supply rate of ink (μl min^{-1})	20-140
Applied voltage (kV)	3.0~20.0
Temperature (°C)	40-60

Table S5. Electromechanical properties of as-deposited glycine/PVP films. For each composition, five samples were used for the measurements and the standard errors were given in the table. The ε_r is calculated from experimentally determined film's capacitance C_x, film's thickness d, and electrode area A: $\varepsilon_r = (C_x \times d)/(\varepsilon_0 \times A)$.

**Films (Ratio of glycine to PVP)**	d_{33} **(pC N^{-1})**	d_{33} **(pm V^{-1})**	ε_r **(@ 100Hz)**	T_c **(℃)**	g_{33} **(10^{-3} VmN^{-1})**	**Young's modulus (MPa)**
1:2.5		1.6±0.96	4.4±0.5	~180		181±27
1:2	1.1±0.1	2.7±0.26	4.2±0.7	~181	~29	241±32
1:1.5	5.1±1.1	8.7±1.6	4.3±0.6	~183	~134	276±42
1:1	7.2±1.0	9.2±1.2	4.3±0.3	~185	~190	308±31
1:0.75	9.1±1.2	10.3±2	4.2±0.7	~185	~244	344±20
1:0.5	9.6±1.4	12.3±2.5	4.3±0.8	~185	~252	441±33

Table S6. Young's modulus of the as-deposited glycine/PVP films compared with some other piezoelectric materials.

**Material**	**Young's modulus (GPa)**
BTO films (*61*)	130
BTO bulk (*61*)	80
PZT(52/48) films (*62*)	126
α-glycine (*42*)	30
β-glycine (*42*)	15
γ-glycine (*42*)	28
PVA/γ-glycine/PVA films (*37*)	4
PVDF films (*63*)	1.5
**β-glycine/PVP films (this work)**	**0.3**

Table S7. List of piezoelectric coefficients of some representative piezoelectric biomaterials, which are plotted in Supplementary Fig. 23.

Material	Piezoelectric coefficient (pm V^{-1})	Measurement method
Wood (*64*)	0.1	Stress-induced electric polarization measurements
Bone (*65*)	0.2	Stress-induced electric polarization measurements
Silk (*66*)	1.5	Stress-induced current measurements
Collagen (*67*)	2.6	PFM
Virus (*68*)	3.9	PFM
Chitin (*69*)	4.0	PFM
γ-glycine-PVA film (*37*)	6	Quasi-static d_{33} meter
β-glycine film (*41*)	11.2	PFM
**β-glycine/PVP film (this work)**	**9.2**	**PFM and d_{33} meter**

Table S8. List of piezoelectric properties of representative piezoelectric films fabricated with different manufacturing techniques, which are plotted in Fig. 3F. Pulsed laser deposition (PLD); Aerosol deposition (AD); Laser interferometric vibrometer (LIV); Double beam laser interferometer (DBLI); Pneumatic loading method (PLM); Piezoresponse force microscopy (PFM); low temperature cofired ceramics (LTCC).

Materials system	Types	g_{33} (10^{-3} VmN^{-1})	T_c (°C)	Reference
$0.95K_{0.6}Na_{0.4}Nb_{0.965}Sb_{0.035}O_{2.98}BaZrO_{2.97}Bi_{0.5}K_{0.5}HfO_3$	KNN-based ceramic	~14	181-192	(*70*)
$0.964K_{0.4}Na_{0.6}Nb_{0.955}Sb_{0.045}O_{0.994}BiFeO_{2.97}Bi_{0.5}Na_{0.5}ZrO_3$	KNN-based ceramic	~23	237	(*71*)
$0.96K_{0.5}Na_{0.5}Nb_{0.96}Sb_{0.04}O_3$-$0.01BaZrO_{2.97}Bi_{0.5}Na_{0.5}ZrO_3$	KNN-based ceramic	~21	252	(*72*)
$0.96K_{0.4}Na_{0.6}Nb_{0.96}Sb_{0.04}O_{2.96}(Bi_{0.45}Sm_{0.05})$ $Na_{0.5}ZrO_3$	KNN-based ceramic	~27	268	(*73*)
$0.95K_{0.525}Na_{0.475}Nb_{0.965}Sb_{0.035}O_3$-0.02 $BaZr_{0.5}Hf_{0.5}O_{2.97}(Bi_{0.5}Na_{0.5})ZrO_3$	KNN-based ceramic	~19	255	(*74*)
$0.955K_{0.48}Na_{0.52}NbO_3$-$0.04Bi_{0.5}Na_{0.5}ZrO_3$-$0.005BiScO_3$	KNN-based ceramic	~22	317	(*75*)
$0.958K_{0.5}Na_{0.5}Nb_{0.975}Sb_{0.25}O_3$-$0.035Bi_{0.5}Na_{0.5}ZrO_{2.993}BiFeO_3$	KNN-based ceramic	~20	269	(*76*)
Commercial PZT (Navy type II; Industry Type 5A)	PZT-based ceramic	23.2	350	(*77*)
Commercial PZT (Navy type V; Industry Type 5J)	PZT-based ceramic	21.3	270	(*77*)
Commercial PZT (Navy type VI; Industry Type 5H)	PZT-based ceramic	19	225	(*77*)
<001>-textured $Pb(Zr_{0.55}Ti_{0.45})O_3$	PZT-based ceramic	100	360	(*78*)
$BaTiO_3$	BTO-based ceramic	11	120	(*79*)
$Ba(Ti_{0.8}Zr_{0.2})O_3$–$(Ba_{0.7}Ca_{0.3})TiO_3$	BTO-based ceramic	22	93	(*80*)
$0.82Ba(Ti_{0.89}Sn_{0.11})O_3$–x0.18$(Ba_{0.7}Ca_{0.3})TiO_3$	BTO-based ceramic	26	60	(*81*)
$0.84BaTiO_3$–$0.16(0.4CaTiO_3$–$0.6BaSnO_3)$	BTO-based ceramic	21	63	(*82*)
Commercial P(VDF-TrFE) (FC 20, Produced from Pizeotech)	Piezo-polymer	282-301	136	
Commercial P(VDF-TrFE) (FC 25, Produced from Pizeotech)	Piezo-polymer	282-301	115	
Commercial P(VDF-TrFE) (FC 30, Produced from Pizeotech)	Piezo-polymer	177-203	100	
Commercial P(VDF-TrFE) (FC 45, Produced from Pizeotech)	Piezo-polymer	177-203	60	
Chitin	Piezo-biomaterial	108	75	(*69*) (*83*) (*84*)
Cellulose	Piezo-biomaterial	27	132	(*85, 86*)

Collagen	Piezo-biomaterial	47	60	(*67*, *87*, *88*)
Virus	Piezo-biomaterial	73	95	(*68*, *89*)
Silk	Piezo-biomaterial	56	178	(*66*, *90*)
β-glycine crystal	Glycine-based material	296	67	(*58*)
γ-glycine/PVA films	Glycine-based material	158	195	(*37*)
**β-glycine/PVP films (This work)**	**Glycine-based material**	**190**	**~185**	

Table S9. The material properties used in FEM Simulations.

Property	Density (kg/m^3)	Sound speed (m/s)	Attenuation (Np/m/MHz)	Specific heat (J/(kg·K))	Thermal conductivity (W/(m·K))
**Tissue phantom**	1044	1568	8.55	3710	0.59
**Human tissue**	1000-1100	1450-1640	4.03-17.27	3600-3890	0.45-0.56

Supplementary Movies

Movie S1: The TEA printing process of a glycine/PVP film.
Movie S2: Solubility test of the glycine/PVP film.
Movie S3: Deliquescence test of the glycine/PVP film.
Movie S4: Simulated acoustic pressure field and the periodic alteration of the electric potential distribution of piezoelectric film.
Movie S5: Periodically change of electric potential distribution within the piezoelectric film.
Movie S6: Wireless signal transmission powered by the Gly-PUEH device ex vivo.

REFERENCES AND NOTES

1. J. Curie, P. Curie, Development by pressure of polar electricity in hemihedral crystals with inclined faces. *Bull. Sco. Min. de France* **3**, 90–93 (1880).

2. B. Jaffe, R. S. Roth, S. Marzullo, Piezoelectric properties of lead zirconate-lead titanate solid-solution ceramics. *J. Appl. Phys.* **25**, 809–810 (1954).

3. H. Kawai, The piezoelectricity of poly (vinylidene fluoride). *Jpn. J. Appl. Phys.* **8**, 975 (1969).

4. S. C. Masmanidis, R. B. Karabalin, I. De Vlaminck, G. Borghs, M. R. Freeman, M. L. Roukes, Multifunctional nanomechanical systems via tunably coupled piezoelectric actuation. *Science* **317**, 780–783 (2007).

5. Q. Zheng, M. Peng, Z. Liu, S. Li, R. Han, H. Ouyang, Y. Fan, C. Pan, W. Hu, J. Zhai, Dynamic real-time imaging of living cell traction force by piezo-phototronic light nano-antenna array. *Sci. Adv.* **7**, eabe7738 (2021).

6. W. Yan, G. Noel, G. Loke, E. Meiklejohn, T. Khudiyev, J. Marion, G. Rui, J. Lin, J. Cherston, A. Sahasrabudhe, Single fibre enables acoustic fabrics via nanometre-scale vibrations. *Nature* **603**, 616–623 (2022).

7. X. Li, Z. Zhang, Z. Peng, X. Yan, Y. Hong, S. Liu, W. Lin, Y. Shan, Y. Wang, Z. Yang, Fast and versatile electrostatic disc microprinting for piezoelectric elements. *Nat. Commun.* **14**, 6488 (2023).

8. Y. Zhao, Y. Gu, B. Liu, Y. Yan, C. Shan, J. Guo, S. Zhang, C. D. Vecitis, G. Gao, Pulsed hydraulic-pressure-responsive self-cleaning membrane. *Nature* **608**, 69–73 (2022).

9. W.-Z. Zeng, K. L. Marshall, S. Min, I. Daou, M. W. Chapleau, F. M. Abboud, S. D. Liberles, A. Patapoutian, PIEZOs mediate neuronal sensing of blood pressure and the baroreceptor reflex. *Science* **362**, 464–467 (2018).

10. D. Kim, S. A. Han, J. H. Kim, J. Lee, S. Kim, S. Lee, Biomolecular piezoelectric materials: From amino acids to living tissues. *Adv. Mater.* **32**, 1906989 (2020).

11. J. Li, Y. Long, F. Yang, X. Wang, Degradable piezoelectric biomaterials for wearable and implantable bioelectronics. *Curr. Opin. Solid State Mater. Sci.* **24**, 100806 (2020).

12. Z. Zhang, S. Liu, Q. Pan, Y. Hong, Y. Shan, Z. Peng, X. Xu, B. Liu, Y. Chai, Z. Yang, Van der Waals exfoliation processed biopiezoelectric submucosa ultrathin films. *Adv. Mater.* **34**, 2200864 (2022).

13. E. Fukada, K. Hara, Piezoelectric effect in blood vessel walls. *J. Physical Soc. Japan* **26**, 777–780 (1969).

14. A. A. Marino, R. O. Becker, Piezoelectricity in hydrated frozen bone and tendon. *Nature* **253**, 627–628 (1975).

15. D. De Rossi, C. Domenici, P. Pastacaldi, Piezoelectric properties of dry human skin. *IEEE Trans. Electr. Insul.* **EI-21**, 511–517 (1986).

16. N. More, G. Kapusetti, Piezoelectric material–A promising approach for bone and cartilage regeneration. *Med. Hypotheses* **108**, 10–16 (2017).

17. E. Fukada, On the piezoelectric effect of silk fibers. *J. Physical Soc. Japan* **11**, 1301A (1956).

18. S. Guerin, A. Stapleton, D. Chovan, R. Mouras, M. Gleeson, C. McKeown, M. R. Noor, C. Silien, F. M. F. Rhen, A. L. Kholkin, Control of piezoelectricity in amino acids by supramolecular packing. *Nat. Mater.* **17**, 180–186 (2018).

19. E. Fukada, S. Sasaki, Piezoelectricity of α-chitin. *J. Polym. Sci. Polym. Phys. Ed.* **13**, 1845–1847 (1975).

20. W. H. Iding, Transducer and method of making same. Google Patents (1971).

21. H. D. Espinosa, R. A. Bernal, M. Minary-Jolandan, A review of mechanical and electromechanical properties of piezoelectric nanowires. *Adv. Mater.* **24**, 4656–4675 (2012).

22. M. Smith, S. Kar-Narayan, Piezoelectric polymers: Theory, challenges and opportunities. *Int. Mater. Rev.* **67**, 65–88 (2022).

23. T.-Y. Zhang, M. Zhao, P. Tong, Fracture of piezoelectric ceramics. *Adv. Appl. Mech.* **38**, 147–289 (2002).

24. M. Reches, E. Gazit, Controlled patterning of aligned self-assembled peptide nanotubes. *Nat. Nanotechnol.* **1**, 195–200 (2006).

25. M. Li, M. Liu, F. Qi, F. R. Lin, A. K.-Y. Jen, Self-assembled monolayers for interfacial engineering in solution-processed thin-film electronic devices: Design, fabrication, and applications. *Chem. Rev.* **124**, 2138–2204 (2024).

26. A. Levin, T. A. Hakala, L. Schnaider, G. J. L. Bernardes, E. Gazit, T. P. J. Knowles, Biomimetic peptide self-assembly for functional materials. *Nat. Rev. Chem.* **4**, 615–634 (2020).

27. B. Yang, D. J. Adams, M. Marlow, M. Zelzer, Surface-mediated supramolecular self-assembly of protein, peptide, and nucleoside derivatives: From surface design to the underlying mechanism and tailored functions. *Langmuir* **34**, 15109–15125 (2018).

28. S. Jia, S. C. Phua, Y. Nihongaki, Y. Li, M. Pacella, Y. Li, A. M. Mohammed, S. Sun, T. Inoue, R. Schulman, Growth and site-specific organization of micron-scale biomolecular devices on living mammalian cells. *Nat. Commun.* **12**, 5729 (2021).

29. J. D. Hartgerink, E. Beniash, S. I. Stupp, Peptide-amphiphile nanofibers: A versatile scaffold for the preparation of self-assembling materials. *Proc. Natl. Acad. Sci. U.S.A.* **99**, 5133–5138 (2002).

30. M. A. Wood, K. A. Ellenbogen, Cardiac pacemakers from the patient's perspective. *Circulation* **105**, 2136–2138 (2002).

31. X. Wang, J. Song, J. Liu, Z. L. Wang, Direct-current nanogenerator driven by ultrasonic waves. *Science* **316**, 102–105 (2007).

32. C. Shi, V. Andino-Pavlovsky, S. A. Lee, T. Costa, J. Elloian, E. E. Konofagou, K. L. Shepard, Application of a sub–0.1-mm^3 implantable mote for in vivo real-time wireless temperature sensing. *Sci. Adv.* **7**, eabf6312 (2021).

33. Y. Hong, L. Jin, B. Wang, J. Liao, B. He, T. Yang, Z. Long, P. Li, Z. Zhang, S. Liu, A wood-templated unidirectional piezoceramic composite for transmuscular ultrasonic wireless power transfer. *Energ. Environ. Sci.* **14**, 6574–6585 (2021).

34. R. Lay, G. S. Deijs, J. Malmström, The intrinsic piezoelectric properties of materials–A review with a focus on biological materials. *RSC Adv.* **11**, 30657–30673 (2021).

35. L. Jiang, Y. Yang, Y. Chen, Q. Zhou, Ultrasound-induced wireless energy harvesting: From materials strategies to functional applications. *Nano Energy* **77**, 105131 (2020).

36. Y. Zheng, Z. Zhang, Y. Zhang, Q. Pan, X. Yan, X. Li, Z. Yang, Enhancing ultrasound power transfer: Efficiency, acoustics, and future directions. *Adv. Mater.* 2407395 (2024).

37. F. Yang, J. Li, Y. Long, Z. Zhang, L. Wang, J. Sui, Y. Dong, Y. Wang, R. Taylor, D. Ni, Wafer-scale heterostructured piezoelectric bio-organic thin films. *Science* **373**, 337–342 (2021).

38. V. Nguyen, R. Zhu, K. Jenkins, R. Yang, Self-assembly of diphenylalanine peptide with controlled polarization for power generation. *Nat. Commun.* **7**, 13566 (2016).

39. E. Seyedhosseini, K. Romanyuk, D. Vasileva, S. Vasilev, A. Nuraeva, P. Zelenovskiy, M. Ivanov, A. N. Morozovska, V. Y. Shur, H. Lu, Self-assembly of organic ferroelectrics by evaporative dewetting: A case of β-glycine. *ACS Appl. Mater. Interfaces* **9**, 20029–20037 (2017).

40. Z. Wang, Z. Zhang, Z. Peng, X. Yang, X. Li, Y. Shan, B. Liu, X. Xu, Y. Gao, Z. Yang, Self-charging and long-term face masks leveraging low-cost, biodegradable and sustainable piezoelectric nanofiber membrane. *Nano Mater. Sci.* 10.1016/j.nanoms.2024.02.012 (2024).

41. Z. Zhang, X. Li, Z. Peng, X. Yan, S. Liu, Y. Hong, Y. Shan, X. Xu, L. Jin, B. Liu, Active self-assembly of piezoelectric biomolecular films via synergistic nanoconfinement and in-situ poling. *Nat. Commun.* **14**, 4094 (2023).

42. V. S. Bystrov, E. Seyedhosseini, I. Bdikin, S. Kopyl, S. M. Neumayer, J. Coutinho, A. L. Kholkin, Bioferroelectricity in nanostructured glycine and thymine: Molecular modeling and ferroelectric properties at the nanoscale. *Ferroelectrics* **475**, 107–126 (2015).

43. D. Vasileva, S. Vasilev, A. L. Kholkin, V. Y. Shur, Domain diversity and polarization switching in amino acid β-glycine. *Materials* **12**, 1223 (2019).

44. M. D. Ward, Perils of polymorphism: Size matters. *Isr. J. Chem.* **57**, 82–92 (2017).

45. J. W. Mullin, Crystal growth. *Crystallization* (2001), pp. 216–288.

46. J. Bernstein, *Polymorphism in Molecular Crystals* (International Union of Crystal, Oxford Univ. Press, 2nd Edn., 2020), vol. 30.

47. D.-R. Chen, D. Y. H. Pui, Experimental investigation of scaling laws for electrospraying: Dielectric constant effect. *Aerosol Sci. Tech.* **27**, 367–380 (1997).

48. A. Jaworek, A. Krupa, Classification of the modes of EHD spraying. *J. Aerosol Sci.* **30**, 873–893 (1999).

49. J. Huang, R. G. Holt, R. O. Cleveland, R. A. Roy, Experimental validation of a tractable numerical model for focused ultrasound heating in flow-through tissue phantoms. *J. Acoust. Soc. Am.* **116**, 2451–2458 (2004).

50. F. Duck, *Physical Properties of Tissues: A Comprehensive Reference Book* (Academic Press, 2013).

51. C. F. Guimarães, L. Gasperini, A. P. Marques, R. L. Reis, The stiffness of living tissues and its implications for tissue engineering. *Nat. Rev. Mater.* **5**, 351–370 (2020).

52. E. S. Hosseini, L. Manjakkal, D. Shakthivel, R. Dahiya, Glycine–chitosan-based flexible biodegradable piezoelectric pressure sensor. *ACS Appl. Mater. Interfaces* **12**, 9008–9016 (2020).

53. J. Sui, J. Li, L. Gu, C. A. Schmidt, Z. Zhang, Y. Shao, E. Gazit, P. U. P. A. Gilbert, X. Wang, Orientation-controlled crystallization of c-glycine films with enhanced piezoelectricity. *J. Mater. Chem. B* **10**, 6958–6964 (2022).

54. J.-H. Lee, K. Heo, K. Schulz-Schönhagen, J. H. Lee, M. S. Desai, H.-E. Jin, S.-W. Lee, Diphenylalanine peptide nanotube energy harvesters. *ACS Nano* **12**, 8138–8144 (2018).

55. K. Romanyuk, V. Slabov, D. Alikin, P. Zelenovskiy, M. R. P. Correia, K. Keller, R. A. S. Ferreira, S. Vasilev, S. Kopyl, A. Kholkin, Piezoactive dense diphenylalanine thin films via solid-phase crystallization. *Appl. Mater. Today* **26**, 101261 (2022).

56. V. Nguyen, K. Jenkins, R. Yang, Epitaxial growth of vertically aligned piezoelectric diphenylalanine peptide microrods with uniform polarization. *Nano Energy* **17**, 323–329 (2015).

57. L. Adler-Abramovich, D. Aronov, P. Beker, M. Yevnin, S. Stempler, L. Buzhansky, G. Rosenman, E. Gazit, Self-assembled arrays of peptide nanotubes by vapour deposition. *Nat. Nanotechnol.* **4**, 849–854 (2009).

58. J. Ryu, C. B. Park, High-temperature self-assembly of peptides into vertically well-aligned nanowires by aniline vapor. *Adv. Mater.* **20**, 3754–3758 (2008).

59. D. Farrar, K. Ren, D. Cheng, S. Kim, W. Moon, W. L. Wilson, J. E. West, S. M. Yu, Permanent polarity and piezoelectricity of electrospun α-helical poly (α-amino acid) fibers. *Adv. Mater.* **23**, 3954–3958 (2011).

60. K. Maruyama, Y. Kawakami, F. Narita, Young's modulus and ferroelectric property of $BaTiO_3$ films formed by aerosol deposition in consideration of residual stress and film thickness. *Jpn. J. Appl. Phys.* **61**, SN1011 (2022).

61. C. J. Ramos-Cano, M. Miki-Yoshida, R. Herrera-Basurto, F. Mercader-Trejo, L. Fuentes-Cobas, O. Auciello, A. Hurtado-Macías, Effect of the orientation polarization and texturing on nano-mechanical and piezoelectric properties of PZT (52/48) films. *Appl. Phys. A* **129**, 113 (2023).

62. M. El Achaby, F. Z. Arrakhiz, S. Vaudreuil, E. M. Essassi, A. Qaiss, Piezoelectric β-polymorph formation and properties enhancement in graphene oxide–PVDF nanocomposite films. *Appl. Surf. Sci.* **258**, 7668–7677 (2012).

63. E. Fukada, Piezoelectricity of wood. *J. Physical Soc. Japan* **10**, 149–154 (1955).

64. E. Fukada, I. Yasuda, On the piezoelectric effect of bone. *J. Physical Soc. Japan* **12**, 1158–1162 (1957).

65. T. Yucel, P. Cebe, D. L. Kaplan, Structural origins of silk piezoelectricity. *Adv. Funct. Mater.* **21**, 779–785 (2011).

66. D. Denning, J. I. Kilpatrick, E. Fukada, N. Zhang, S. Habelitz, A. Fertala, M. D. Gilchrist, Y. Zhang, S. A. M. Tofail, B. J. Rodriguez, Piezoelectric tensor of collagen fibrils determined at the nanoscale. *ACS Biomater Sci. Eng.* **3**, 929–935 (2017).

67. B. Y. Lee, J. Zhang, C. Zueger, W.-J. Chung, S. Y. Yoo, E. Wang, J. Meyer, R. Ramesh, S.-W. Lee, Virus-based piezoelectric energy generation. *Nat. Nanotechnol.* **7**, 351–356 (2012).

68. K. Kim, M. Ha, B. Choi, S. H. Joo, H. S. Kang, J. H. Park, B. Gu, C. Park, C. Park, J. Kim, Biodegradable, electro-active chitin nanofiber films for flexible piezoelectric transducers. *Nano Energy* **48**, 275–283 (2018).

69. K. Xu, J. Li, X. Lv, J. Wu, X. Zhang, D. Xiao, J. Zhu, Superior piezoelectric properties in potassium–sodium niobate lead-free ceramics. *Adv. Mater.* **28**, 8519–8523 (2016).

70. B. Wu, H. Wu, J. Wu, D. Xiao, J. Zhu, S. J. Pennycook, Giant piezoelectricity and high Curie temperature in nanostructured alkali niobate lead-free piezoceramics through phase coexistence. *J. Am. Chem. Soc.* **138**, 15459–15464 (2016).

71. J. Ma, B. Wu, W. Wu, M. Chen, Structure and electrical properties of (0.99− x) $K_{0.5}Na_{0.5}Nb_{0.96}Sb_{0.04}O_3$–$0.01BaZrO_{3\text{-}x}Bi_{0.5}Na_{0.5}ZrO_3$ lead-free piezoelectric ceramics. *J. Mater. Sci. Mater. Electron.* **29**, 12323–12329 (2018).

72. C. Shi, J. Ma, J. Wu, X. Wang, F. Miao, Y. Huang, K. Chen, W. Wu, B. Wu, Coexistence of excellent piezoelectric performance and high Curie temperature in KNN-based lead-free piezoelectric ceramics. *J. Alloys Compd.* **846**, 156245 (2020).

73. C. Shi, J. Ma, J. Wu, K. Chen, B. Wu, $(Bi_{0.5}Na_{0.5})ZrO_3$ modified KNN-based ceramics: Enhanced electrical properties and temperature insensitivity. *Ceram. Int.* **46**, 2798–2804 (2020).

74. L. Jiang, Z. Tan, L. Xie, Y. Li, J. Xing, J. Wu, Q. Chen, D. Xiao, J. Zhu, Study of the relationships among the crystal structure, phase transition behavior and macroscopic properties

of modified (K, Na) NbO_3-based lead-free piezoceramics. *J. Eur. Ceram. Soc.* **38**, 2335–2343 (2018).

75. B. Wu, J. Ma, W. Wu, M. Chen, Improved piezoelectricity in ternary potassium–sodium niobate lead-free ceramics with large strain. *J. Mater. Chem. C* **8**, 2838–2846 (2020).

76. MIDE, PIEZO.COM Materials Technical Data, (2020), p. 1; https://info.piezo.com/hubfs/Data-Sheets/piezo-material-properties-data-sheet-20201112.pdf.

77. J. Li, W. Qu, J. Daniels, H. Wu, L. Liu, J. Wu, M. Wang, S. Checchia, S. Yang, H. Lei, Lead zirconate titanate ceramics with aligned crystallite grains. *Science* **380**, 87–93 (2023).

78. R. Bechmann, Elastic, piezoelectric, and dielectric constants of polarized barium titanate ceramics and some applications of the piezoelectric equations. *J. Acoust. Soc. Am.* **28**, 347–350 (1956).

79. W. Liu, X. Ren, Large piezoelectric effect in Pb-free ceramics. *Phys. Rev. Lett.* **103**, 257602 (2009).

80. C. Zhao, H. Wu, F. Li, Y. Cai, Y. Zhang, D. Song, J. Wu, X. Lyu, J. Yin, D. Xiao, Practical high piezoelectricity in barium titanate ceramics utilizing multiphase convergence with broad structural flexibility. *J. Am. Chem. Soc.* **140**, 15252–15260 (2018).

81. L.-F. Zhu, B.-P. Zhang, L. Zhao, J.-F. Li, High piezoelectricity of $BaTiO_3$–$CaTiO_3$–$BaSnO_3$ lead-free ceramics. *J. Mater. Chem. C* **2**, 4764–4771 (2014).

82. T. Mitra, G. Sailakshmi, A. Gnanamani, S. T. K. Raja, T. Thiruselvi, V. M. Gowri, N. V. Selvaraj, G. Ramesh, A. B. Mandal, Preparation and characterization of a thermostable and biodegradable biopolymers using natural cross-linker. *Int. J. Biol. Macromol.* **48**, 276–285 (2011).

83. D. Saravanan, K. Vijayalakshmi, T. Gomathi, P. N. Sudha, Physicochemical characterization of nylon fiber reinforced chitosan composites. *Pharm. Lett.*, **6**, 139–145 (2014)

84. K. M. Picker, S. W. Hoag, Characterization of the thermal properties of microcrystalline cellulose by modulated temperature differential scanning calorimetry. *J. Pharm. Sci.* **91**, 342–349 (2002).

85. Y. García, Y. B. Ruiz-Blanco, Y. Marrero-Ponce, C. M. Sotomayor-Torres, Orthotropic piezoelectricity in 2D nanocellulose. *Sci. Rep.* **6**, 34616 (2016).

86. E. Marzec, K. Pietrucha, The effect of different methods of cross-linking of collagen on its dielectric properties. *Biophys. Chem.* **132**, 89–96 (2008).

87. S. Muszyński, M. Kwiecień, M. Świetlicki, P. Dobrowolski, J. Tatarczak, B. Gładyszewska, Effects of replacing soybean meal with chickpea seeds in the diet on mechanical and thermal properties of tendon tissue in broiler chicken. *Poult. Sci.* **97**, 695–700 (2018).

88. S. D. Branston, E. C. Stanley, J. M. Ward, E. Keshavarz-Moore, Determination of the survival of bacteriophage M13 from chemical and physical challenges to assist in its sustainable bioprocessing. *Biotechnol. Bioprocess Eng.* **18**, 560–566 (2013).

89. X. Hu, Q. Lu, D. L. Kaplan, P. Cebe, Microphase separation controlled β-sheet crystallization kinetics in fibrous proteins. *Macromolecules* **42**, 2079–2087 (2009).
